# ChatGPT models provide higher‐quality but lower‐readability responses than Google Gemini regarding anterior shoulder instability, with no added benefit of the orthopaedic expert plugin

**DOI:** 10.1002/ksa.70255

**Published:** 2025-12-26

**Authors:** Khaled Skaik, Sean Omoseni, Danielle Dagher, Darshil Shah, Theodorakys Marín Fermín, Piero Agostinone, Ashraf Hantouly, Moin Khan

**Affiliations:** ^1^ Faculty of Medicine & Health Sciences McGill University Montreal Quebec Canada; ^2^ School of Medicine University of Edinburgh Edinburgh Scotland UK; ^3^ Division of Orthopaedic Surgery McMaster University Hamilton Ontario Canada; ^4^ Santa Sofía Hospital Caracas Venezuela; ^5^ Biomedical and Neuromotor Sciences Department University of Bologna Bologna Italy

**Keywords:** anterior shoulder instability, artificial intelligence, ChatGPT, large language models, shoulder stabilization surgery

## Abstract

**Purpose:**

The purpose is to analyze and compare the quality and readability of information regarding anterior shoulder instability and shoulder stabilization surgery from three LLMs: ChatGPT 4o, ChatGPT Orthopaedic Expert (OE) and Google Gemini.

**Methods:**

ChatGPT 4o, ChatGPT OE and Google Gemini were used to answer 21 commonly asked questions from patients on anterior shoulder instability. The responses were independently rated by three fellowship‐trained orthopaedic surgeons using the validated Quality Analysis of Medical Artificial Intelligence (QAMAI) tool. Assessors were blinded to the model, and evaluations were performed twice, 3 weeks apart. Readability was measured using Flesch Reading Ease Score (FRES) and Flesch–Kincaid Grade Level (FKGL). This study adhered to TRIPOD‐LLM. Statistical analysis included the Friedman test, the Wilcoxon signed‐rank tests and inter‐class coefficients.

**Results:**

Inter‐rater reliability between three surgeons was good or excellent reliability in all LLMs. ChatGPT OE and ChatGPT 4o demonstrated comparable overall performance, each achieving a median QAMAI score of 22 with interquartile ranges (IQRs) of 5.25 and 6.75, respectively, with median (IQR) domain scores for accuracy 4 (1) and 4 (1), clarity 4 (1) and 4 (1), relevance 4 (1) and 4 (1), completeness 4 (1) and 4 (1), provision of sources 1 (0) for both and usefulness 4 (1) and 4 (1), respectively. Google Gemini showed lower scores across these domains (accuracy 3 [1], clarity 3 [1], relevance 3 [1.25], completeness 3 [0.25], sources 3 [3] and usefulness 3 [1.25]), with a median QAMAI score of 19 (5.25) (*p* < 0.01 vs. each ChatGPT model). Readability was higher for Google Gemini (FRES = 36.96, FKGL = 11.92) than for ChatGPT OE (FRES = 21.90, FKGL = 14.94) and ChatGPT 4o (FRES = 24.24, FKGL = 15.11), indicating easier‐to‐read content (*p* < 0.01). There was no significant difference between ChatGPT 4o and OE in overall quality or readability.

**Conclusions:**

ChatGPT 4o and ChatGPT OE provided statistically higher‐quality responses than Google Gemini, though all models showed good‐quality responses overall. However, responses generated by ChatGPT 4o and OE were more difficult to read than those generated by Google Gemini.

**Level of Evidence:**

Level V, expert opinion.

**Abbreviations**
ACL: anterior cruciate ligament
AI: artificial intelligence
ASI: anterior shoulder instability
ChatGPT OE: ChatGPT Orthopaedic Expert
CI: confidence interval
FKGL: Flesch–Kincaid Grade Level
FRES: Flesch Reading Ease Score
IBM SPSS: International Business Machines Statistical Package for the Social Sciences
ICC: intraclass correlation coefficient
IRR: inter‐rater reliability
LLM: large language model
QAMAI: Quality Assessment of Medical AI
TRIPOD‐LLM: Transparent Reporting of a Multivariable Model for Individual Prognosis or Diagnosis –Large Language Model

## INTRODUCTION

Artificial intelligence (AI) has been shown to have increasing applications in patient education and clinical decision‐making [19, 32]. As the popularity of AI chatbots increases, the gradual shift from users relying on search engines (Google, Bing, etc.) for their most pressing questions shifts in favour of generative AI services, also referred to as large language models (LLMs), such as ChatGPT (OpenAI) and Google Gemini [22]. More recently, newly developed models such as DeepSeek have emerged after the inception and widespread adoption of ChatGPT [30].

The earliest orthopaedic literature on AI described LLMs as promising yet risky tools, highlighting concerns around accuracy, plagiarism and responsible use [13, 29]. Whereas information is abundant and readily available through LLMs and search engines, both tools may be susceptible to misinformation in orthopaedics [58]. This is especially true for earlier‐generation models and in general‐purpose LLMs that have not been fine‐tuned for medical or domain‐specific applications [3, 24, 48]. The conversational manner in which these models communicate has highlighted the potential for their use in facilitating patient education in healthcare [32].

Anterior shoulder instability (ASI) is a relatively common orthopaedic condition that requires a thorough assessment and targeted management [18]. It is particularly prevalent among individuals who participate in contact or overhead sports, where repetitive stress or traumatic dislocations frequently occur [40, 45, 46]. Furthermore, older adults may also develop ASI as a result of traumatic injuries or degenerative changes [21, 39, 47]. Given its impact across a wide range of age groups and activity levels, it is essential to examine the key sources of information that patients rely on when confronted with musculoskeletal problems [18]. Previous studies have assessed the accuracy of LLMs in the context of ASI by comparing their responses to expert consensus statements and by evaluating their answers to frequently asked patient questions (FAQ) related to shoulder stabilization surgery [4, 23]. However, these investigations have mainly focused on ChatGPT 4.0 and have not examined how its performance compares to other LLMs [4, 23]. This may be due to the limited availability of other models during the time these studies were conducted.

ChatGPT 4o is one of the latest multimodal models in the ChatGPT family and has only recently been evaluated in a limited orthopaedic context, including spine surgery [52], hip arthroscopy [6], patellar instability [51] and anterior cruciate ligament (ACL) injuries [20]. As LLM capabilities expand, advanced features and plugins have been introduced to enhance medical information access [25]. For instance, one study used ChatGPT 4o's deep research mode and DeepSeek‐R1's DeepThink function to evaluate responses to ACL‐related FAQs [20].

Recently, ChatGPT became available with an orthopaedic expert plugin, referred to as ChatGPT Orthopaedic Expert (ChatGPT OE), which is trained by orthopaedic specialists and has over 5000 conversations [10]. Despite its popularity, this plugin has not yet been evaluated for its ability to answer orthopaedic‐related FAQs.

The authors of this study opted to specifically evaluate ChatGPT models and Google Gemini as they remain the two most commonly used LLMs worldwide in 2025, with an estimated 557 million users for ChatGPT and 70.1 million for Google Gemini [31].

This study aims to compare the quality of information in answering questions related to ASI provided by ChatGPT 4o, ChatGPT OE and Google Gemini in terms of accuracy, comprehensiveness, clinical relevance and readability. Given that ChatGPT OE is trained on domain‐specific orthopaedic data [10] and may produce more technical and specialized language than the general‐purpose models, it was hypothesized that ChatGPT OE would provide higher‐quality responses and would be less readable than ChatGPT 4o and Google Gemini.

## METHODS

### Question pool development

Institutional review board approval was not required for this study as it did not involve human subjects. A pool of commonly asked questions about ASI and shoulder stabilization surgery was gathered from international consensus statements and established institution web pages [23, 37]. A final set of 21 questions was carefully selected to be queried to the three LLMs by two fellowship‐trained orthopaedic experts (A.H. and M.K.) based on those most clinically relevant for real‐world clinical practice, breadth of coverage across FAQs and feasibility of evaluators. No formal priori power calculation was performed, given the nature of this exploratory comparative study. The pooled questions are displayed in Table 1.

**Table 1:** Questions pooled and posed to three different large language models.

Questions
1. What causes anterior shoulder instability?
2. Who is at risk of developing anterior shoulder instability?
3. What is a Bankart lesion?
4. How can anterior shoulder instability be prevented?
5. If I've dislocated my shoulder once, am I more likely to dislocate it again?
6. What are the symptoms of anterior shoulder instability?
7. How is anterior shoulder instability diagnosed?
8. What are the potential long‐term effects of anterior shoulder instability?
9. When is surgery necessary for anterior shoulder instability?
10. What are the non‐surgical treatment options for anterior shoulder instability?
11. Does inflammation play a role in managing anterior shoulder instability? If so, for how long?
12. What are the surgical treatment options for anterior shoulder instability?
13. What are the risks and potential complications of anterior shoulder instability surgery?
14. What is the likelihood of recurrence after anterior shoulder instability treatment?
15. What is a Bankart repair?
16. What is a Latarjet procedure?
17. What is a Remplissage procedure?
18. What are the indications and contraindications for Bankart repair?
19. What are the indications and contraindications for the Latarjet procedure?
20. What is the expected recovery time after surgery for anterior shoulder instability?
21. When can I return to sports and drive after anterior shoulder instability treatment?

The questions were posed to all three LLMs on 11 March 2025, with no additional clarifications or follow‐up questions. All LLMs had no preexisting memory regarding the queries posed and were given the same prompts with no word count restrictions to better simulate a real‐world scenario in which a patient asks questions to an expert.

### Quality assessment

Three fellowship‐trained orthopaedic surgeons (D.S., T.M.F. and R.A.), who were uninvolved in the question pool development, independently assessed the responses using the evidence‐based rating system described by Vaira et al., which has been applied in existing literature, called the Quality Analysis of Medical Artificial Intelligence (QAMAI) tool (Table 2) [9, 38, 50, 55]. Responses were ranked on a 5‐point Likert scale: ‘*strongly disagree*’ (1 point), ‘*disagree*’ (2 points), ‘*neutral*’ (3 points), ‘*agree*’ (4 points) or ‘*strongly agree*’ (5 points) on each of the following categories: accuracy, clarity, relevance, completeness, provision of sources and references, and usefulness [50]. Numerical scores were assigned to each category and then summed up to give the total QAMAI score according to Table 3 [50]. The surgeons were blinded to the model being evaluated, and assessments were conducted on two separate occasions 3 weeks apart to minimize recall bias and ensure consistency in scoring.

**Table 2:** Domains and definitions of the Quality Analysis of Medical Artificial Intelligence tool scoring system.

Domain	Definition
Accuracy	The information provided is accurate and up to date.
Clarity	The answer is clear and comprehensible in terms of language and scientific terminology.
Relevance	The information provided is relevant and directly answer to the question posed.
Completeness	The response adequately covers all aspects of the question and provides sufficient information, including areas of uncertainty.
Provision of sources and references	The response provides reliable sources and references to support the health information presented.
Usefulness	The response provides information to meet the user's health information needs.

**Table 3:** The Quality Analysis of Medical Artificial Intelligence (AI) tool scoring system.

Score	Classification	Description
6–11 points	Poor quality	The AI system provides information that is largely unreliable or incomplete. Immediate improvement is required
12–17 points	Fair quality	The AI system provides some useful information, but there are significant areas for improvement
18–23 points	Good quality	The AI system provides mostly reliable and complete information, but there may be some areas for refinement
24–29 points	Very good quality	The AI system provides reliable and complete information in most areas. There are minor areas for improvement
30 points	Excellent quality	The AI system provides highly reliable and complete information

### Readability assessment

**Figure 1:**
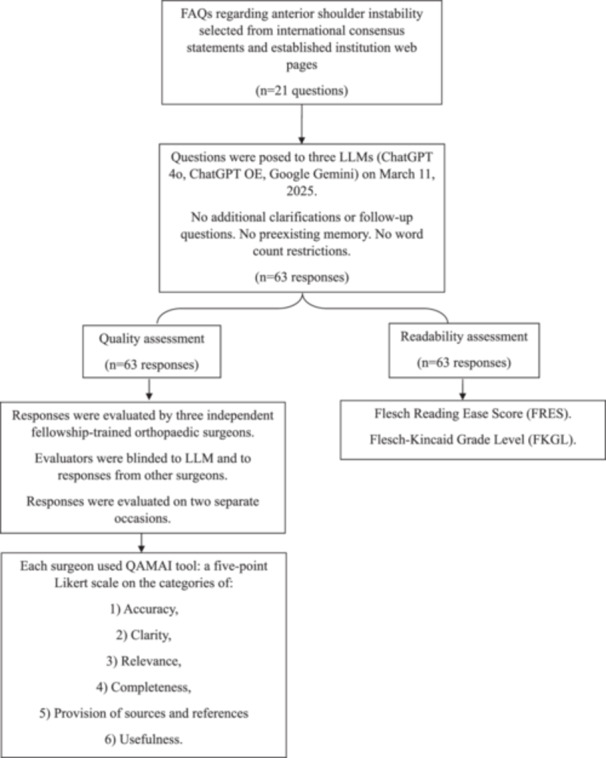
Flowchart of methodology used in evaluating responses regarding anterior shoulder instability. FAQ, frequently asked questions; LLMs, large language models; QAMAI, Quality Analysis of Medical Artificial Intelligence.

The readability of each response was also analyzed using the Flesch Reading Ease Score (FRES) and the Flesch–Kincaid Grade Level (FKGL) on a Microsoft Word document (Microsoft 365) [8, 16, 55]. The FRES assigns a score from 0 (*extremely difficult to read*) to 100 (*very easy to read*). The FKGL translates readability into an approximate US grade level, ranging from first grade to college level, with higher scores indicating more complex to read. Both metrics account for factors such as average word syllables and sentence length [8, 16]. A flowchart of the methodology is provided in Figure 1.

### TRIPOD‐LLM

To ensure transparency and methodological rigour in reporting, this study adheres to the recently developed Transparent Reporting of a multivariable model for Individual Prognosis or Diagnosis–Large Language Models (TRIPOD‐LLM) guidelines [17]. TRIPOD‐LLM is an extension of the TRIPOD+AI framework designed specifically to address the unique challenges of evaluating LLMs in healthcare research. It consists of a comprehensive checklist of 19 main items and 50 subitems that guide standardized reporting across all stages of LLM research, from model description and prompt design to evaluation methods, performance assessment, and discussion of bias, transparency and human oversight [17].

### Data analysis

Descriptive statistics, including median, interquartile range, proportions, and 95% confidence intervals (CIs), were calculated using Microsoft Excel (Microsoft 365, Microsoft Corporation). The Friedman test was used as an overall non‐parametric test to identify differences between models with subsequent pairwise testing with the Wilcoxon signed‐rank test comparing each domain and the overall score of the QAMAI, as well as readability differences between the three LLMs, with statistical significance set at *p* < 0.05 (IBM SPSS, IBM Corp.). Bonferroni correction was performed as well as effect sizes (*r*) calculated. The inter‐rater reliability (IRR) was calculated for the QAMAI scores at a level using the intraclass correlation coefficient (ICC), where values below 0.5 indicate poor reliability, between 0.5 and 0.75 moderate reliability, between 0.75 and 0.9 good reliability, and any value above 0.9 indicates excellent reliability [33].

## RESULTS

### IRR

For ratings of Google Gemini responses, the ICC for the overall QAMAI score was 0.94 (95% CI = 0.88–0.97), indicating excellent reliability. For ChatGPT OE and ChatGPT 4o, the ICCs were 0.85 (95% CI = 0.74–0.93) and 0.82 (95% CI = 0.70–0.92), respectively, both indicating good reliability.

### Quality assessment

The questions and answers provided by each LLM are presented in Table S1. This study adhered to TRIPOD‐LLM guidelines [17] (Supporting Information S2). The overall highest ranked response was the response to Question 6 ‘What are the symptoms of anterior shoulder instability?’ given by ChatGPT 4o. In contrast, the lowest‐ranked response was the response to Question 15, ‘What is a Bankart repair?’ given by Google Gemini (Supporting Information S3). Importantly, all the responses given by ChatGPT 4o and ChatGPT OE did not provide references in their responses, receiving a score of 1 (*strongly disagree*) for provision of resources and references in the QAMAI tool (Table 4).

**Table 4:** Median scores and interquartile ranges (IQRs) for each category in the Quality of Medical Artificial Intelligence tool for each large language model (LLM).

LLM	Accuracy (median, IQR)	Clarity (median, IQR)	Relevance (median, IQR)	Completeness (median, IQR)	Provision of sources and reference (median, IQR)	Usefulness (median, IQR)	Overall median (IQR)
ChatGPT 4o	4 (0.5)	4 (2)	4 (1.5)	4 (2.25)	1 (0)	4 (1.25)	22 (6.75)
Google Gemini	3 (1)	3 (1)	3 (1.25)	3 (0.25)	3 (3)	3 (1.25)	19 (5.25)
ChatGPT Orthopaedic Expert	4 (0.25)	4 (1.25)	4 (2)	4 (1)	1 (0)	4 (1.25)	22 (5.25)

### Friedman and Wilcoxon tests

The differences between all three LLMs were significant in Friedman's test (*p* < 0.001). Applying a Bonferroni correction for six comparisons (three evaluators at two separate occasions) (adjusted *α* symbol = 0.0083) did not alter these findings, and all results remained significant. Additional paired Wilcoxon signed‐rank tests between models with Bonferroni correction (*α* = 0.017) resulted in continued significance between respective domains. Similar patterns were observed across the domains excluding provision of sources with large effect sizes (*r* = 0.55–0.74) when comparing either ChatGPT OE or ChatGPT 4o to Google Gemini, and small effect sizes when comparing ChatGPT 4o to ChatGPT OE (*r* = 0.01–0.08) (Table 5).

**Table 5:** Friedman test comparing scores between all three models, and the Wilcoxon signed‐rank test comparing score pairs.

	Statistical test	Accuracy	Clarity	Relevance	Completeness	Provision of sources and references	Usefulness	Overall
All three LLMs	Friedman	<0.001	<0.001	<0.001	<0.001	<0.001	<0.001	<0.001
ChatGPT OE vs. Google Gemini^a^	Wilcoxon	<0.01	<0.01	<0.01	<0.01	<0.01	<0.01	<0.01
ChatGPT 4o vs. Google Gemini^a^	Wilcoxon	<0.01	<0.01	<0.01	<0.01	<0.01	<0.01	<0.01
ChatGPT OE vs. ChatGPT 4o^b^	Wilcoxon	0.58	0.39	0.41	0.90	No difference	0.42	0.71

Abbreviations: LLM, large language model; OE, orthopaedic expert.

^a^ Large effect size (*r* correlation coefficient).

^b^ Small effect size.

Both ChatGPT OE and ChatGPT 4o demonstrated comparable performance across all 21 questions, including identical scores for provision of sources (median = 1, IQR = 0). ChatGPT OE achieved a median overall QAMAI score of 22 (IQR = 5.25). ChatGPT 4o showed similar performance with a median overall QAMAI score of 22 (IQR 6.75), indicating that both models provided good‐quality information (Table 4). In contrast, Google Gemini had lower median scores: accuracy 3 (IQR = 1), clarity 3 (IQR = 1), relevance 3 (IQR = 1.25), completeness 3 (IQR = 0.25), provision of sources 3 (IQR = 3) and usefulness 3 (IQR = 1.25). However, the overall mean QAMAI score of 19 (IQR = 5.25), which still indicates good quality information (Table 4).

**Figure 2:**
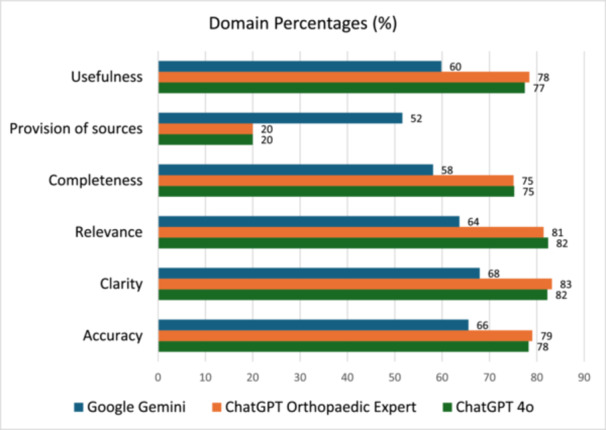
Domain percentages across the three large language models.

Google Gemini responses were ranked to have statistically worse QAMAI scores compared to each ChatGPT 4o and ChatGPT OE (*p* < 0.01). However, there was no statistical difference between ChatGPT 4o and ChatGPT OE (Table 5). ChatGPT 4o and OE consistently achieved higher percentages across all QAMAI domains (range = 77–83%) compared to Google Gemini (range = 58–68%), except in the provision of sources category, where Google Gemini led with 52% versus 20% for both ChatGPT models (Figure 2).

**Figure 3:**
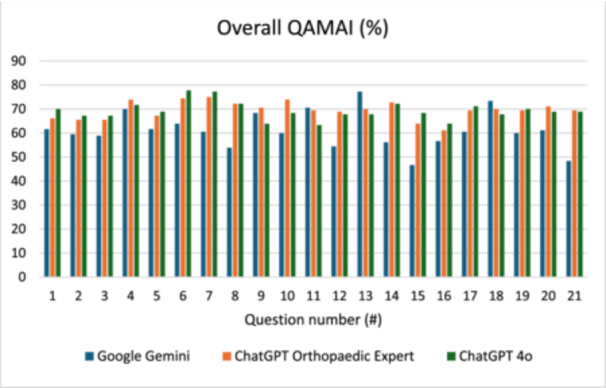
Overall scores of Quality Analysis of Medical Artificial Intelligence (QAMAI) tool across included questions.

**Figure 4:**
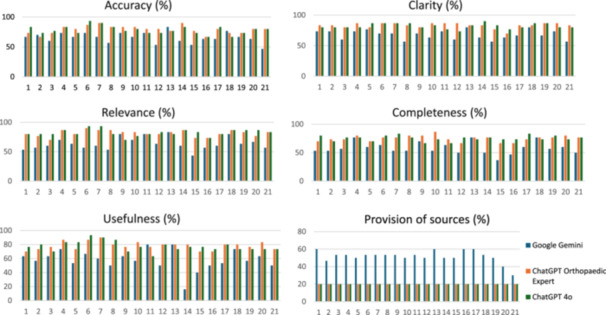
Domain‐specific performance across all 21 questions answered by the three large language models.

When analyzed at the question and domain‐specific level, Questions 6 and 7 received the highest overall quality scores for both ChatGPT 4o (78% and 77%) and ChatGPT OE (74% and 75%), outperforming Google Gemini (64% and 61%) (Figure 3). These stronger results were driven primarily by higher accuracy, relevance and usefulness (Figure 4). In contrast, the lowest scores were observed for Questions 15 and 16, with ChatGPT 4o scoring 68% and 64%, ChatGPT OE scoring 68% and 61% and Google Gemini scoring 47% and 57%, respectively. ChatGPT OE outperformed ChatGPT 4o on Question 10 (74% vs. 68%), while their performance was similar across the remaining questions.

### Readability assessment

**Figure 5:**
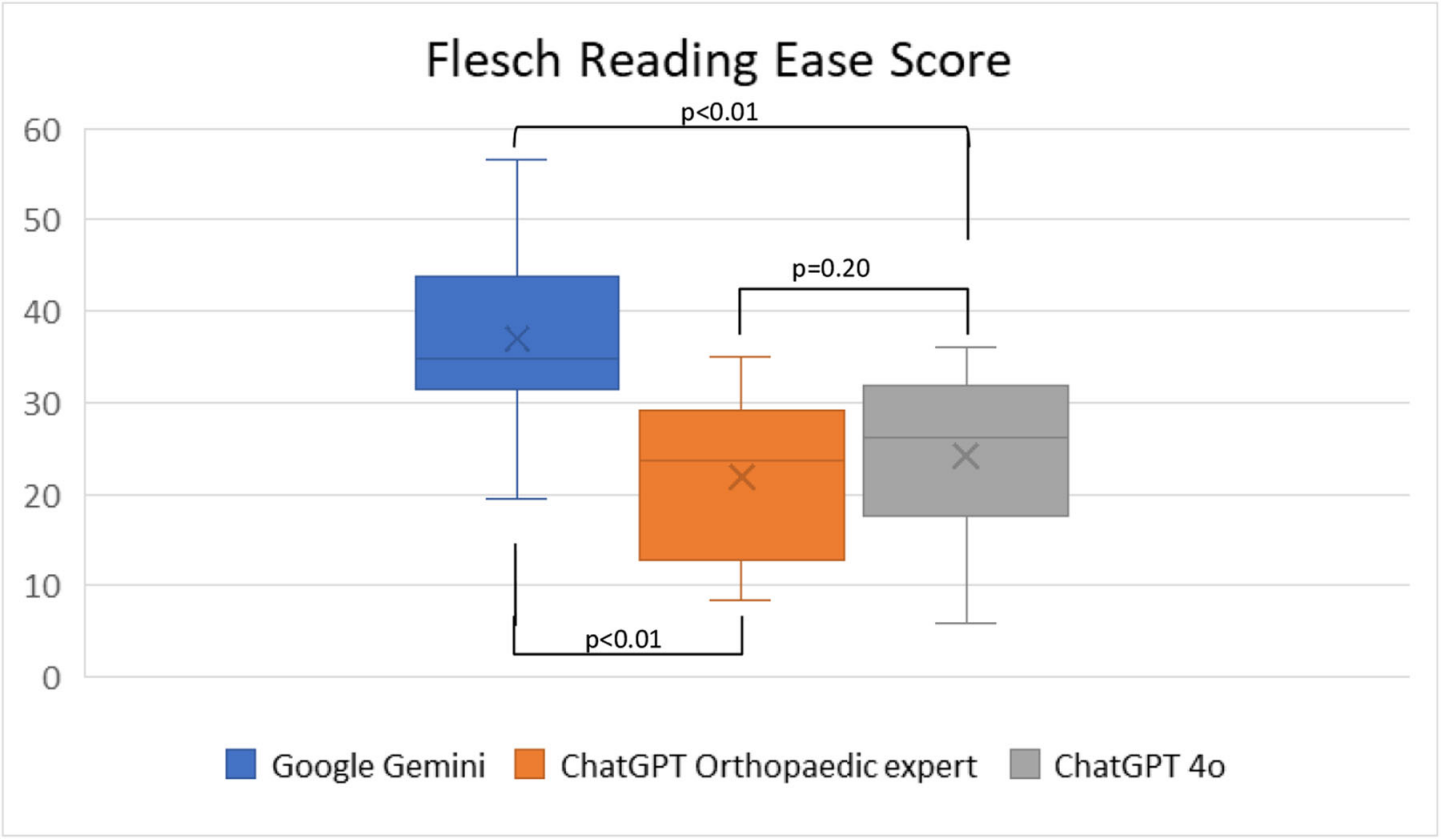
Flesch Reading Ease Score for three large language models.

**Figure 6:**
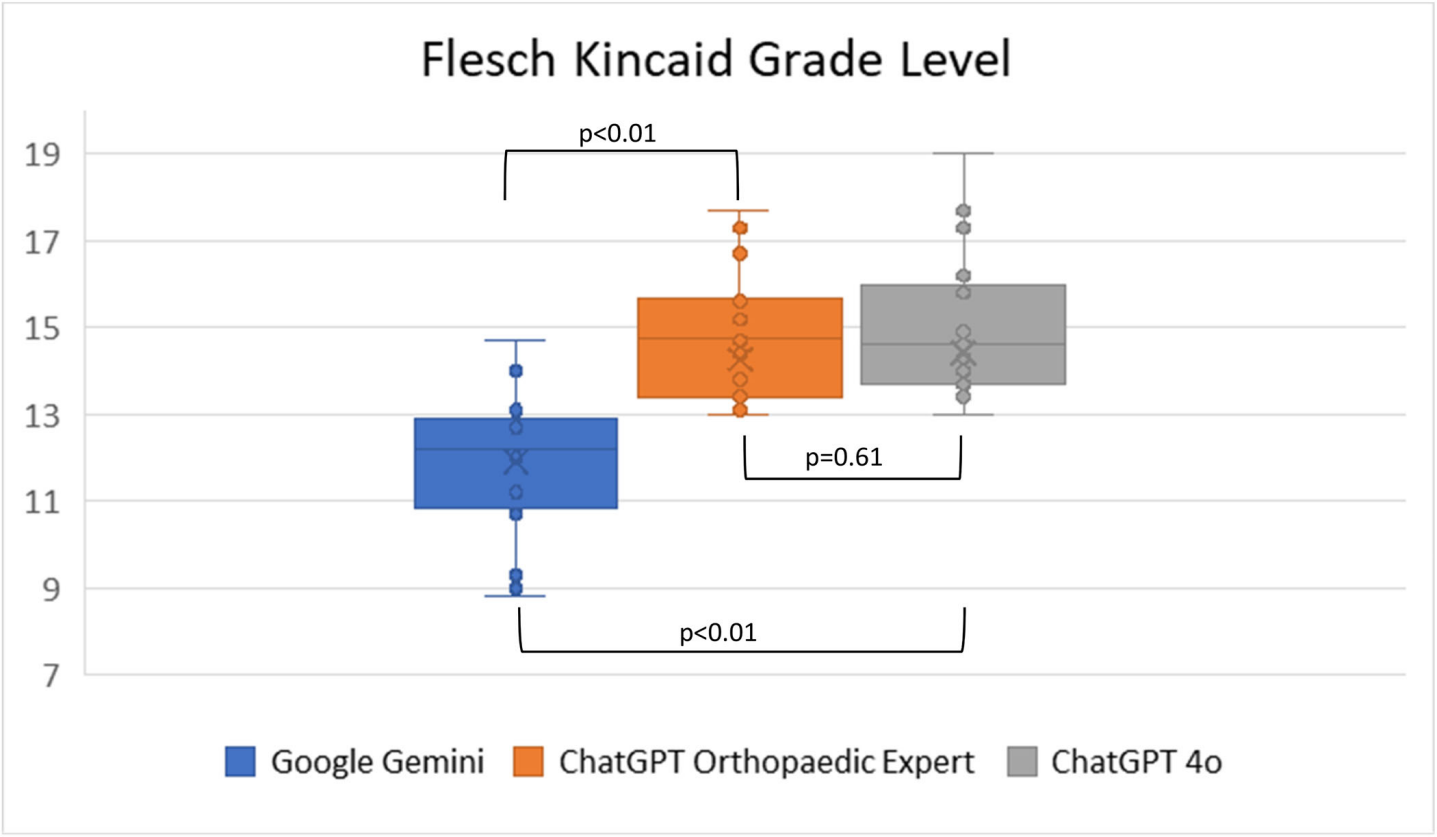
Flesch Kincaid Grade Level scores for three large language models.

The mean FRES for Google Gemini was 36.96 (range = 19.6–56.5), which corresponds to text that is difficult to read, best understood by college students. The mean FKGL was 11.92 (range = 8.8–14.7), indicating content written at a 12th‐grade reading level. For ChatGPT OE, the mean FRES was 21.90 (range = 8.5–35.1), classified as very difficult to read, appropriate for university graduates, and the mean FKGL was 14.94 (range = 13.0–17.7), corresponding to a college‐level reading grade. For ChatGPT 4o, the mean FRES was 24.24 (range = 5.9–36.0), also indicating very difficult to read material, and the mean FKGL was 15.11 (range = 13.0–19.0), indicating content at a college or graduate reading level. Google Gemini demonstrated significantly higher FRES and lower FKGL scores compared to each ChatGPT 4o and ChatGPT OE (*p* < 0.01), indicating that its responses were easier to read. However, there was no significant difference in FRES (*p* = 0.20) or FKGL (*p* = 0.60) scores between ChatGPT 4o and ChatGPT OE (Figures 5 and 6).

## DISCUSSION

The main findings of this study are that all three LLMs provided overall good quality information on ASI, as measured by the QAMAI tool, meaning that three AI systems provide reliable and complete information in most areas, but that there are some areas for improvement. While all models achieved scores within the same ‘good quality’ category, both ChatGPT 4o and ChatGPT OE delivered responses of significantly higher quality than Google Gemini, with no added benefit of the Orthopaedic Expert plugin. In contrast, Google Gemini's responses were significantly easier to read than those of either ChatGPT model. Furthermore, the responses generated by ChatGPT 4o and ChatGPT OE did not include references or indicate their sources, whereas Google Gemini consistently provided cited references.

To our knowledge, this is the first to assess the impact of the Orthopaedic Expert plugin within ChatGPT. Additionally, this is the first study to directly compare the performance of Google Gemini, ChatGPT 4o and ChatGPT OE in addressing queries related to ASI. In addition, it is the first study to report its outcomes using the TRIPOD‐LLM checklist [17]. Given the growing number of publications evaluating the performance of LLMs, particularly in patient education in orthopaedics, the authors strongly recommend incorporating the TRIPOD‐LLM framework in future studies to ensure transparency, methodological rigour and standardized reporting [17].

The strongest performance for both ChatGPT 4o and ChatGPT OE was observed on questions about the likelihood of shoulder dislocation (Question 5) and the symptoms of ASI (Question 6), whereas the lowest scores occurred for surgical questions describing the Bankart repair and Latarjet procedures (Questions 15 and 16). This suggests that LLMs may perform better on general, non‐surgical topics than on more technical surgical content, which may be better answered by surgeons.

Notably, ChatGPT OE consistently outperformed ChatGPT 4o on Question 10 as it offered a more comprehensive overview of non‐surgical treatments, including functional training, cryotherapy and patient selection guidance. However, ChatGPT 4o and OE performed similarly on the remaining questions. This may be because ChatGPT 4o, even without a plugin, has already been trained on a broad and detailed foundation of medical knowledge [7]. It is capable of generating high‐quality explanations about orthopaedic conditions, procedures and treatment options on its own. The plugin does not appear to significantly alter the output, and that might be because it simply provides an alternate pathway to access the same type of information. This is consistent with prior work showing minimal performance differences between earlier ChatGPT versions (3.5 vs. 4.0) [1, 2, 34, 36], further supporting the conclusion that different ChatGPT versions often deliver similar overall quality.

However, utilizing ChatGPT's Deep Research function may further enhance response quality [44]. In contrast to conventional ChatGPT 4o prompting, which relies solely on its pre‐trained internal knowledge, the Deep Research feature conducts real‐time online searches and integrates information from external medical journals, clinical guidelines and reputable health resources [44]. By retrieving and integrating current evidence directly into its output, this feature can address gaps in baseline model knowledge and improve accuracy [44]. Supporting this, a recent study evaluating Deep Research for ACL‐surgery FAQs found that it produced responses with high accuracy, consistency and comprehensiveness [20].

Previous studies have examined responses from earlier versions of ChatGPT in orthopaedic surgery, but the accuracy and quality of the information provided to patients remain inconsistent and without a clear consensus [15, 26, 27, 28, 35, 41, 54, 56, 57]. For instance, Wright et al. found that while ChatGPT 3.5's answers were as accurate as information found online, it fell short as a primary information source for total hip and knee arthroplasty, highlighting its limitations in patient education for these procedures [54]. Similarly, Johns et al. found that most of the ChatGPT 3.5‐generated responses were outdated and failed to provide an adequate foundation for patients' understanding regarding their injury and treatment options regarding ACL reconstruction [27]. In contrast, one study found that ChatGPT 3.5 provided clear and mostly accurate information regarding total ankle arthroplasty [5].

In the context of ASI, Hurley et al. assessed ChatGPT 3.5's responses to 23 questions about shoulder stabilization surgery using *Journal of the American Medical Association* (JAMA) and DISCERN scores by three orthopaedic surgery residents. They reported a score of 0, indicating no cited sources, and a DISCERN score of 60, indicating good overall quality. This aligns with our findings, as ChatGPT 4o and OE also scored poorly for source attribution (score of 1) while still achieving a QAMAI median score of 22, indicating good quality [23]. Our study builds on this by identifying the specific domains within QAMAI in which newer ChatGPT models perform best.

In addition, it is important to note that all three LLMs were categorized as providing good‐quality information within the QAMAI framework. However, this three‐point difference (ChatGPT models' median score of 22 and Google Gemini's median score of 19) reflects meaningful reductions in accuracy, clarity, relevance, completeness and usefulness in Google Gemini's responses. Google Gemini achieved a higher subscore only in the provision of references, which elevated its overall QAMAI rating despite the lower quality of the content itself. These limitations may weaken patient confidence, restrict shared decision making and increase the likelihood of misunderstanding important clinical concepts. This concern is amplified by the fact that Google Gemini's responses were easier to read, which may lead patients to confidently understand and rely on information that is less accurate, potentially influencing their decisions inappropriately.

Although the overall quality supports the use of ChatGPT as a supplementary educational tool, it is not sufficiently reliable to replace an expert surgeon's explanation or to serve as a primary information source. This aligns with another study that examined the similarity between GPT‐4.0's responses and expert consensus statements on the diagnosis, non‐operative management and Bankart repair for ASI, which found limited agreement with expert recommendations [4].

Studies evaluating ChatGPT 4o have consistently shown high performance across orthopaedic applications [6, 51, 52]. One study assessed its responses to 20 hip arthroscopy FAQs using a 5‐point Likert scale across relevance, accuracy, clarity and completeness, and reported strong results in all domains, with mean scores of 4.49 for relevance, 4.51 for accuracy, 4.51 for clarity and 4.46 for completeness [6]. These findings align with our results, which also demonstrate high accuracy, clarity, relevance and completeness for ASI.

Following the introduction of ChatGPT 4o, a smaller and faster version, ChatGPT 4o mini, was released to provide a more cost‐efficient alternative [43]. In a study evaluating 21 lumbar disc herniation FAQs, both 4o and 4o mini achieved similarly high accuracy scores (4.65 vs. 4.63; *p* = 0.77), although completeness was significantly higher with the full 4o model (4.72 vs. 4.57; *p* = 0.04) [52]. These results suggest that the efficiency of 4o mini comes at the cost of reduced comprehensiveness, and that later versions of ChatGPT, particularly ChatGPT 4o, consistently outperform earlier models such as ChatGPT 3.5 [43].

In a study comparing multiple LLMs on patellar instability questions reviewed by eight fellowship‐trained sports surgeons, ChatGPT 4o achieved a median Mika score of 2 [38], which indicates a satisfactory response requiring only minimal clarification [51]. It also tied with Claude2 for the highest proportion of Mika 1 responses (47.5%), which represent excellent answers requiring no further clarification. ChatGPT 4o outperformed PerplexityAI and markedly surpassed Google Gemini, which more frequently generated responses requiring moderate (Mika 3) or substantial (Mika 4) clarification [51]. Similar findings have been reported in other areas, such as glucocorticoid‐induced osteoporosis, where ChatGPT 3.5 and 4.0 significantly outperformed Google Gemini in quality [49].

Our study also found that Google Gemini produced more readable responses than the ChatGPT models. This finding is consistent with another study in answering questions regarding developmental dysplasia of the hip [42], about ankle sprains [11] and about patellar instability [51]. However, even the more readable responses from Google Gemini still mostly require a Grade 12 reading level, which limits accessibility for many patients. This high readability demand further supports the view that LLMs should not be relied upon as primary patient‐education resources, as the readability of patient education materials should be no greater than a sixth‐grade reading level [12, 14, 53].

While both ChatGPT and Google Gemini generally provided high‐quality responses, it remains crucial to acknowledge the potential drawbacks of using LLMs. These include the risk of generating inaccurate information, potential biases and discrimination, limited transparency and reliability, cybersecurity concerns and broader ethical and societal implications [7]. Surgeons should be aware of these findings before recommending their use to patients.

To build on these findings, future research should evaluate LLMs using more extensive and diverse question sets across various orthopaedic domains. It should also conduct multilingual or cross‐cultural readability studies to assess accessibility for non‐English‐speaking populations. Tracking model performance over time as AI systems are updated can help determine how improvements or regressions affect clinical relevance and accuracy. Additionally, hybrid evaluation frameworks that combine expert clinician review with patient feedback may offer a more comprehensive approach to assessing the real‐world utility of AI‐generated educational content.

### Limitations

This study does not come without limitations. First, only 21 questions were evaluated, which is a relatively small sample that might contribute to low statistical power. However, this increases the feasibility of the assessments across multiple models, and the number of questions evaluated in this study is still larger than that found in previous studies [26, 28, 35, 41, 54, 59]. In addition, the assessment process is further complicated by its subjective nature. Because the accuracy and quality of ChatGPT's and Google Gemini's responses depend on individual evaluators' judgments, this introduces variability that makes it harder to universally validate the findings. However, inter‐ and intra‐rater reliability assessments were conducted to mitigate this limitation, with the results showing consistent ratings among surgeons. Finally, because LLMs are regularly updated, future outputs may vary. As such, our findings reflect a snapshot of performance from the specific model versions tested and may not generalize to subsequent iterations.

## CONCLUSION

ChatGPT 4o, ChatGPT OE and Google Gemini provided overall good quality responses related to ASI. However, ChatGPT 4o and OE showed statistically higher results in the quality of the responses compared to those generated by Google Gemini, with no difference between the two ChatGPTs. However, the responses generated by ChatGPT were more difficult to read than those generated by Google Gemini. These results highlight the promise of LLMs for improving patient education, but they also emphasize the necessity of careful use and continuous assessment.

## AUTHOR CONTRIBUTIONS

All authors contributed to the study design, data collection, analysis and manuscript preparation.

## CONFLICT OF INTEREST STATEMENT

The authors declare no conflicts of interest.

## ETHICS STATEMENT

Institutional review board approval was not required for this study as it did not involve human subjects.

## Supporting information

Supplemental Material S1. Responses given by each Large Language Model.

Supplemental Material S2. TRIPOD‐LLM Checklist for assessment of Large Language Models.

Supplemental Material S3. Raw data of Quality Analysis of Medical Artificial Intelligence tool scores for each Large Language Model given by each evaluator.

## Data Availability

The data that support the findings of this study are available online in the Supporting Information of this article.
