# Supplementary material for: ChatGPT models provide higher‐quality but lower‐readability responses than Google Gemini regarding anterior shoulder instability, with no added benefit of the orthopaedic expert plugin
Source: Knee Surg Sports Traumatol Arthrosc. 2025 Dec 26;34(2):763–75. doi: 10.1002/ksa.70255 (PMC12850548; doi:10.1002/ksa.70255)
Supplement: Supplementary file 1 — Supplemental Material S1. Responses given by each Large Language Model. [file KSA-34-763-s001.docx]

**Supplemental Table S1.** Questions posed to each LLM alongside their responses.

| **Question** | **ChatGPT 4o** | **ChatGPT OE** | **Google Gemini** |
| --- | --- | --- | --- |
| What causes anterior shoulder instability? | Anterior shoulder instability occurs when the head of the humerus (upper arm bone) repeatedly slips out of the front of the glenoid (the shallow socket in the scapula). This instability can result from various causes, including:  **1. Trauma (Shoulder Dislocation)**   - A common cause of anterior shoulder instability is a traumatic event, such as a fall or sports injury, that forces the arm into an extreme external rotation and abduction (arm raised and rotated outward). This can cause the head of the humerus to dislocate or sublux (partially dislocate) from the front of the socket. - A common result of this trauma is a **Bankart lesion**, which is a tear of the labrum (a cartilage ring that deepens the shoulder socket).   **2. Repetitive Overhead Movements**   - Athletes involved in overhead sports (e.g., baseball, volleyball, swimming) are prone to developing anterior shoulder instability due to the repetitive stresses placed on the shoulder joint. - Over time, this can lead to microtrauma, stretching, and weakening of the ligaments and capsule at the front of the shoulder, resulting in instability.   **3. Capsular and Ligamentous Laxity**   - Some individuals naturally have looser ligaments and capsules, making them more prone to shoulder instability without a significant traumatic event. - This is often referred to as **multidirectional instability** but may still present with anterior instability as the most prominent symptom.   **4. Glenoid Bone Loss**   - In cases of recurrent dislocations or subluxations, part of the glenoid bone can be damaged or worn down (referred to as **glenoid bone loss**). This reduces the stability of the socket and increases the risk of further dislocations.   **5. Hill-Sachs Lesion**   - When the humeral head dislocates anteriorly, it may impact the edge of the glenoid, causing a compression fracture of the humeral head known as a **Hill-Sachs lesion**. This can exacerbate instability by making it easier for the humeral head to slip out of the socket again.   **6. Congenital or Developmental Factors**   - Some individuals may have a shoulder joint with structural differences, such as a shallow glenoid or underdeveloped stabilizing muscles, leading to increased vulnerability to anterior instability.   **7. Weakness of Rotator Cuff Muscles**   - The rotator cuff muscles help stabilize the shoulder joint. Weakness or dysfunction in these muscles can reduce the dynamic stabilization they provide, leading to increased risk of anterior shoulder instability.   In summary, anterior shoulder instability is most commonly caused by traumatic injury, but repetitive overhead activities, ligament laxity, and anatomical variations also play key roles. | Anterior shoulder instability occurs when the structures that stabilize the shoulder joint are weakened or injured, leading to the head of the humerus (upper arm bone) sliding or dislocating forward out of the glenoid (shoulder socket). Several factors contribute to this condition:   1. **Trauma or Injury**: A significant trauma, such as falling on an outstretched arm or experiencing a direct blow to the shoulder, is the most common cause. This often results in a dislocation or subluxation (partial dislocation) of the shoulder joint. 2. **Repetitive Overhead Movements**: Athletes involved in sports like baseball, swimming, or tennis, where the shoulder undergoes repetitive overhead motions, are at higher risk for anterior shoulder instability. This is due to the gradual stretching and weakening of the ligaments and muscles that stabilize the joint. 3. **Labral Tears**: The labrum is a cartilage ring that deepens the shoulder socket. A tear in the labrum, particularly the **Bankart lesion** (a tear in the lower part of the labrum), often occurs after an initial dislocation, making future dislocations more likely. 4. **Ligament Laxity or Hypermobility**: Some individuals have naturally loose or overstretched ligaments (joint laxity), leading to instability. This may be genetic or due to overstretching from certain activities. 5. **Weakness in Rotator Cuff Muscles**: The rotator cuff muscles help keep the shoulder joint stable. Weakness in these muscles can contribute to instability, making the shoulder more prone to dislocation or subluxation. 6. **Capsular Damage**: The joint capsule, a fibrous tissue surrounding the shoulder, can become stretched or torn due to injury or overuse, leading to instability.   Managing anterior shoulder instability may involve physical therapy to strengthen the shoulder muscles, bracing, or, in severe cases, surgical intervention to repair torn ligaments or the labrum. | This is for informational purposes only. For medical advice or diagnosis, consult a professional.  Anterior shoulder instability occurs when the head of the humerus (upper arm bone) dislocates or subluxes (partially dislocates) from the glenoid fossa (shoulder socket).([ref](https://www.ncbi.nlm.nih.gov/books/NBK459125/)) This can be caused by a traumatic injury, such as a fall or sports injury, or by repetitive overuse of the shoulder joint. ([ref](https://www.massgeneral.org/orthopaedics/sports-medicine/conditions-and-treatments/shoulder-instability#:~:text=A%20dislocation%20is%20most%20common,weaken%20the%20muscles%2C%20ligaments%20and))  There are several risk factors that can increase the likelihood of developing anterior shoulder instability, including:   - Age: Younger people are more likely to experience anterior shoulder instability than older people. - Sex: Men are more likely to experience anterior shoulder instability than women. ([ref](https://www.ncbi.nlm.nih.gov/books/NBK538234/)) - Sports: Athletes who participate in contact sports or overhead sports, such as baseball, volleyball, and tennis, are at increased risk. ([ref](https://pmc.ncbi.nlm.nih.gov/articles/PMC8447104/#:~:text=Athletes%20are%20particularly%20prone%20to,shoulder%20to%20be%20in%20a)) - Previous shoulder injuries: People who have had a previous shoulder dislocation or subluxation are at increased risk of developing anterior shoulder instability. - Loose ligaments: Some people are born with naturally loose ligaments in their shoulder joint, which can make them more susceptible to instability.  ([ref](https://www.hopkinsmedicine.org/health/conditions-and-diseases/shoulder-instability#:~:text=Genetic%20Condition,a%20loose%20or%20spacious%20capsule).))   The most common symptom of anterior shoulder instability is pain in the shoulder joint. Other symptoms may include:   - A feeling of the shoulder "giving way" or "popping out" of place - Clicking or snapping in the shoulder joint - Decreased range of motion in the shoulder  ([ref](https://www.londonarmdoc.com/anterior-shoulder-instability-shoulder-surgeon-london/)) - Weakness in the shoulder  ([ref](https://www.nhslanarkshire.scot.nhs.uk/services/physiotherapy-msk/shoulder-instability/#:~:text=There%20is%20a%20chance%20your,stiffness%2C%20giving%20way%20and%20pain.))   If you are experiencing any of these symptoms, it is important to see a doctor to get a diagnosis and treatment plan. Treatment for anterior shoulder instability typically involves a combination of rest, physical therapy, and medication. In some cases, surgery may be necessary to repair damaged ligaments or tendons.  ([ref](https://www.londonarmdoc.com/anterior-shoulder-instability-shoulder-surgeon-london/))  Here are some tips for preventing anterior shoulder instability:   - Warm up properly before participating in any physical activity. - Use proper technique when throwing or lifting objects. - Strengthen the muscles around your shoulder joint. - Avoid activities that put excessive stress on your shoulder joint.   ([ref](https://www.physio-pedia.com/Anterior_Shoulder_Instability)) - If you have a history of shoulder instability, wear a shoulder brace during physical activity.   If you have any further questions, please do not hesitate to ask. |
| **Who is at risk of developing anterior shoulder instability?** | Certain groups of people are at higher risk of developing anterior shoulder instability, often due to specific factors like activity level, occupation, or inherent joint characteristics. Those at increased risk include:  **1. Athletes Engaged in Overhead Sports**   - **Throwing athletes** (e.g., baseball pitchers, quarterbacks) are especially prone to anterior shoulder instability because of the repetitive overhead motion that puts stress on the shoulder joint. - **Swimmers, tennis players, and volleyball players** also engage in repetitive overhead movements, which can lead to overuse of the shoulder joint and stretching of the ligaments, increasing the risk of instability.   **2. Young, Active Individuals**   - **Younger people**, particularly those under 30, are more prone to anterior shoulder dislocations and recurrent instability. This is due to their higher levels of physical activity and the fact that their soft tissues may heal with less tightness after a dislocation. - Individuals in their **teens and early 20s**, especially those involved in contact sports, are at the highest risk of developing recurrent anterior shoulder instability after an initial dislocation.   **3. Contact Sports Participants**   - **Football, rugby, wrestling, and hockey players** are at a higher risk due to the physical nature of these sports, where impacts and falls are common. Direct trauma or force to the shoulder joint can lead to dislocations or subluxations.   **4. Individuals with Joint Laxity or Hypermobility**   - **People with loose or hypermobile joints**, sometimes due to conditions like **Ehlers-Danlos syndrome** or generalized ligamentous laxity, are at increased risk for anterior shoulder instability. Their ligaments and connective tissues are more flexible and less able to stabilize the joint, making dislocations more likely.   **5. People with a History of Shoulder Dislocations**   - Individuals who have had an anterior shoulder dislocation are at a high risk of recurrent dislocations and chronic instability. This is particularly true for younger individuals (under 30), where the recurrence rate is higher.   **6. Occupations Involving Overhead Work**   - **Workers who perform repetitive overhead tasks**, such as painters, electricians, and construction workers, may develop shoulder instability over time due to repetitive strain on the shoulder joint.   **7. Individuals with Glenoid or Humeral Head Defects**   - People who have suffered **glenoid bone loss** (damage to the socket) or have a **Hill-Sachs lesion** (a dent in the humeral head) from a previous dislocation are at higher risk of recurrent anterior shoulder instability due to the reduced stability of the joint.   **8. Weightlifters or Bodybuilders**   - **Heavy lifters**, especially those doing exercises that involve lifting weights overhead (such as military presses or snatches), are also at risk due to the strain placed on the shoulder joint and surrounding tissues.   **9. Aging Population with Degenerative Changes**   - Although younger individuals are more likely to develop anterior shoulder instability, **older adults** with degenerative changes to the shoulder joint (such as rotator cuff degeneration or weakened joint tissues) may also experience instability, especially after traumatic events.   In summary, those at greatest risk of developing anterior shoulder instability include athletes engaged in overhead and contact sports, individuals with joint hypermobility, people with prior shoulder injuries, and those whose activities involve repetitive overhead movements. | Anterior shoulder instability occurs when the shoulder joint repeatedly dislocates or feels unstable due to excessive movement of the humeral head out of the glenoid cavity in the forward direction. Certain individuals and groups are at higher risk of developing this condition:  **1. Athletes**   - **Contact Sports**: Athletes involved in sports like football, rugby, or wrestling are at a higher risk due to the forceful impacts and falls, which can lead to dislocations. - **Overhead Sports**: Sports that require repetitive overhead movements (e.g., swimming, baseball pitching, tennis) can strain the shoulder joint, making it more susceptible to instability. - **Throwing Athletes**: Repeated throwing motions cause stress on the anterior shoulder capsule and ligaments, potentially weakening them over time.   **2. People with Previous Shoulder Dislocations**   - Once the shoulder has dislocated, there’s an increased risk of recurrent dislocations due to stretched or damaged ligaments, cartilage (such as a **Bankart lesion**), or labral tears.   **3. Individuals with Ligamentous Laxity**   - People who are **hypermobile** or have generalized joint laxity (e.g., individuals with Ehlers-Danlos Syndrome or Marfan Syndrome) are at higher risk because their ligaments are naturally looser and less stable.   **4. Young Adults (18–25 years)**   - The majority of traumatic anterior shoulder dislocations occur in young adults, especially males. This group tends to engage in high-energy activities that can lead to shoulder injuries.   **5. People with Poor Shoulder Muscle Strength**   - Weak or imbalanced muscles around the shoulder joint, especially the rotator cuff and scapular stabilizers, may lead to poor joint stability, increasing the risk of anterior shoulder instability.   **6. Manual Laborers**   - People involved in heavy or repetitive overhead work (e.g., construction workers, mechanics) are prone to shoulder injuries and chronic instability due to repetitive stress on the joint.   Preventive measures, such as strengthening the shoulder muscles, improving proprioception, and modifying risky activities, can help reduce the risk for these groups. | This is for informational purposes only. For medical advice or diagnosis, consult a professional.  Anterior shoulder instability, the most common type of shoulder instability, occurs when the head of the humerus (upper arm bone) slips out of the glenoid (shoulder socket) in a forward direction. ([ref](https://pmc.ncbi.nlm.nih.gov/articles/PMC6243418/)) This can happen due to a traumatic injury, such as a fall or sports injury, or it can develop over time due to repetitive overhead activities.  **Factors that can increase your risk of developing anterior shoulder instability include:**   - **Age:** Younger people are more likely to experience a shoulder dislocation, and they are also more likely to experience recurrent instability. ([ref](https://www.ncbi.nlm.nih.gov/books/NBK459125/)) - **Sex:** Men are more likely to experience shoulder dislocations than women. ([ref](https://my.clevelandclinic.org/health/diseases/17746-dislocated-shoulder#:~:text=Men%20are%20more%20than%20twice,dislocate%20a%20shoulder%20as%20women.)) - **Sports participation:** Athletes who participate in contact sports, such as football and rugby, or overhead sports, such as baseball and volleyball, are at increased risk of shoulder dislocations. ([ref](https://blog.orthoindy.com/2015/09/14/the-danger-of-shoulder-dislocations-in-contact-sports/)) - **Previous shoulder injury:** People who have had a previous shoulder dislocation are at increased risk of recurrent instability.  ([ref](https://ijspt.scholasticahq.com/article/116278-risk-factors-associated-with-first-time-and-recurrent-shoulder-instability-a-systematic-review#:~:text=Male%20sex%2C%20age%20%3C30%20years,risk%20associated%20with%20recurrent%20shoulder)) - **Ligament laxity:** People with naturally loose ligaments in their shoulders are more likely to experience instability. ([ref](https://orthoinfo.aaos.org/en/diseases--conditions/chronic-shoulder-instability/))   **Additional risk factors:**   - **Anatomical variations:** Some people have anatomical variations in their shoulder joint that can make them more susceptible to instability.  ([ref](https://orthoinfo.aaos.org/en/diseases--conditions/chronic-shoulder-instability/)) - **Connective tissue disorders:** People with certain connective tissue disorders, such as Ehlers-Danlos syndrome, may have increased joint laxity, making them more prone to shoulder instability. ([ref](https://www.nhs.uk/conditions/ehlers-danlos-syndromes/#:~:text=types%20of%20EDS-,Hypermobile%20EDS,joint%20pain%20and%20clicking%20joints))   If you have any of these risk factors, you may want to talk to your doctor about ways to prevent shoulder instability.  **Here are some tips to help prevent shoulder instability:**   - **Strengthen the muscles around your shoulder joint.** - **Avoid activities that put excessive stress on your shoulder joint.**   ([ref](https://www.massgeneral.org/orthopaedics/sports-medicine/conditions-and-treatments/shoulder-instability)) - **Wear protective gear when participating in contact sports.**   ([ref](https://pmc.ncbi.nlm.nih.gov/articles/PMC9808839/#:~:text=Nevertheless%2C%20the%20use%20of%20bracing,for%20shoulder%20instability%2C%20such%20as)) - **If you have a previous shoulder injury, follow your doctor's instructions for rehabilitation.**   If you experience any symptoms of shoulder instability, such as pain, clicking, or popping in your shoulder, or a feeling that your shoulder is slipping out of joint, see your doctor for diagnosis and treatment. Early diagnosis and treatment can help prevent further damage to your shoulder and improve your long-term outcome. |
| **What is a Bankart lesion?** | A **Bankart lesion** is a specific type of injury to the shoulder joint, particularly to the **labrum**, which is a ring of cartilage that surrounds the glenoid (the socket of the shoulder joint). This lesion typically occurs when the **anterior part of the labrum** is torn, often following a **shoulder dislocation**, especially when the dislocation happens in the forward (anterior) direction.  **Key points:**   - **Location**: It involves a tear in the lower part of the glenoid labrum. - **Cause**: Most commonly caused by **anterior shoulder dislocations**, which are frequently the result of trauma, such as falling on an outstretched arm or a direct blow to the shoulder. - **Effect**: When the labrum is torn in this way, the stability of the shoulder is compromised, increasing the risk of recurrent dislocations.   **Symptoms of a Bankart Lesion:**   - **Recurrent shoulder dislocations** or instability - **Pain** during shoulder movement - A sensation of the shoulder "giving way" or feeling loose - **Weakness** or limited range of motion in the shoulder   **Treatment:**   - **Non-surgical**: Physical therapy to strengthen the muscles around the shoulder and improve stability. - **Surgical**: In cases of recurrent dislocations or failure of conservative treatments, surgery may be required to repair the torn labrum, often through arthroscopic procedures.   A Bankart lesion is often discussed in conjunction with **Hill-Sachs lesions**, which are injuries to the humeral head (ball of the shoulder joint) that often accompany anterior dislocations. | A **Bankart lesion** is an injury to the **anterior-inferior part of the glenoid labrum**, a fibrous ring that surrounds the shoulder socket (glenoid) to help stabilize the shoulder joint. This injury typically occurs when the shoulder dislocates anteriorly (forward), causing the labrum to tear away from the bone.  Bankart lesions are most commonly associated with **recurrent shoulder dislocations**, particularly in younger, active individuals. The injury compromises the stability of the shoulder joint, making future dislocations more likely.  **Symptoms of a Bankart lesion may include:**   - **Recurrent shoulder dislocations** - **Shoulder instability** (feeling like the shoulder may "give out" during movement) - **Pain** or discomfort, particularly with overhead or rotational movements - **Decreased range of motion** or weakness in the shoulder   **Diagnosis:**  A **physical examination** is typically followed by imaging studies, such as an **MRI** or **arthroscopy**, to confirm the presence of a Bankart lesion.  **Treatment:**   - **Non-surgical treatment**: Involves rest, physical therapy, and strengthening exercises, particularly for less severe cases. - **Surgical repair**: This may be necessary for recurrent dislocations or when conservative treatment fails. The surgeon will reattach the torn labrum to restore stability to the shoulder.   Rehabilitation following surgery involves **physical therapy** and **gradual strengthening** to restore full function and prevent further instability. | A Bankart lesion is an injury to the glenoid labrum, a ring of cartilage that surrounds the shoulder socket. ([ref](https://orthoinfo.aaos.org/en/diseases--conditions/shoulder-joint-tear-glenoid-labrum-tear/#:~:text=The%20head%20of%20the%20upper%20arm%20bone%20is%20usually%20much,the%20socket%20to%20help%20stabilize))  It often occurs as a result of a shoulder dislocation. ([ref](https://pmc.ncbi.nlm.nih.gov/articles/PMC9165262/#:~:text=The%20proportion%20of%20Hill%E2%80%93Sachs,59%25)%20and%20this%20was))    **Causes**   - Shoulder dislocation: The most common cause is a shoulder dislocation, where the humerus (upper arm bone) is forced out of the glenoid (shoulder socket). ([ref](https://www.hopkinsmedicine.org/health/conditions-and-diseases/shoulder-dislocation#:~:text=What%20is%20a%20shoulder%20dislocation,out%20of%20your%20shoulder%20joint.)) - Repetitive overhead motions: In some cases, repetitive overhead motions can also lead to a Bankart lesion.   ([ref](https://www.ncbi.nlm.nih.gov/books/NBK587359/))   **Symptoms**   - Pain: Pain in the shoulder, especially with overhead activities or at night.  ([ref](https://my.clevelandclinic.org/health/diseases/bankart-lesion-glenoid-labrum-tear)) - Instability: A feeling of looseness or instability in the shoulder.   ([ref](https://www.ncbi.nlm.nih.gov/books/NBK587359/#:~:text=The%20Bankart%20lesion%20was%20first,instability%20of%20the%20glenohumeral%20joint.)) - Limited range of motion: Difficulty moving the shoulder through its full range of motion.   ([ref](https://my.clevelandclinic.org/health/diseases/bankart-lesion-glenoid-labrum-tear)) - Mechanical symptoms: Clicking, popping, or catching sensations in the shoulder.  ([ref](https://www.coastalorthoteam.com/blog/what-is-a-bankart-lesion-signs-symptoms-and-repair))   **Diagnosis**   - Physical examination: A doctor will examine the shoulder and assess its range of motion and stability. ([ref](https://pmc.ncbi.nlm.nih.gov/articles/PMC5124608/#:~:text=Diagnosis%20of%20a%20Bankart%20lesion%20requires%20a%20detailed%20assessment%20of,by%20shoulder%20instability%20(3).)) - Imaging tests: X-rays or MRI scans may be used to confirm the diagnosis and rule out other injuries.   ([ref](https://www.ncbi.nlm.nih.gov/books/NBK587359/))   **Treatment**   - Non-surgical treatment: In some cases, rest, ice, and physical therapy may be enough to heal a Bankart lesion. ([ref](https://pmc.ncbi.nlm.nih.gov/articles/PMC6534104/#:~:text=Generally%2C%20Bankart%20lesions%20are%20managed,or%20recurrent%20dislocation%20%5B7%5D.)) - Surgery: If non-surgical treatment is not effective, surgery may be necessary to repair the torn labrum.   ([ref](https://my.clevelandclinic.org/health/diseases/bankart-lesion-glenoid-labrum-tear#:~:text=This%20is%20sometimes%20called%20a,Bankart%20repair%20by%20shoulder%20arthroscopy.))   **Prognosis**  The prognosis for a Bankart lesion is generally good with appropriate treatment. ([ref](https://my.clevelandclinic.org/health/diseases/bankart-lesion-glenoid-labrum-tear#:~:text=Outlook%20%2F%20Prognosis,-What's%20the%20outlook&You%20may%20or%20may%20not,the%20outlook%20afterward%20is%20good.)) Most people are able to return to their normal activities within a few months of surgery.  **Prevention**   - Strengthening the muscles around the shoulder can help to prevent shoulder dislocations and Bankart lesions. ([ref](https://www.uhdb.nhs.uk/shoulder-instabilitydislocation/#:~:text=Physiotherapy%20exercises%20help%20to%20strengthen,muscles%20and%20ligaments%20to%20ensure)) - Avoiding repetitive overhead motions can also help to reduce the risk of injury.  ([ref](https://www.simphysio.com.au/blog/shoulder-disolocations))   If you are experiencing any of the symptoms of a Bankart lesion, it is important to see a doctor for diagnosis and treatment. Early diagnosis and treatment can help to improve your prognosis and prevent further injury. ([ref](https://pubmed.ncbi.nlm.nih.gov/38709911/#:~:text=Early%20diagnosis%20and%20treatment%20are,glenoid%20bone%20loss%20can%20occur.)) |
| **How can anterior shoulder instability be prevented?** | Preventing anterior shoulder instability, especially in individuals at risk (such as athletes involved in contact sports or those with a history of dislocations), involves strengthening the shoulder muscles, improving joint stability, and using proper techniques during physical activities. Here are some key strategies to prevent anterior shoulder instability:  **1. Strengthening the Rotator Cuff Muscles:**   - The **rotator cuff** provides dynamic stability to the shoulder joint, so strengthening these muscles (supraspinatus, infraspinatus, teres minor, and subscapularis) can help maintain joint stability. - Exercises such as **internal and external rotation** with resistance bands, and shoulder abduction, help build strength and endurance in the rotator cuff.   **2. Strengthening the Scapular Stabilizers:**   - The muscles that stabilize the **scapula** (shoulder blade), such as the **serratus anterior, trapezius, rhomboids, and levator scapulae**, play a crucial role in overall shoulder mechanics. - Strengthening exercises like **scapular retraction, protraction, and depression movements** (such as rows, push-ups plus, and scapular pinches) help maintain the shoulder’s proper alignment and prevent instability.   **3. Improving Proprioception and Neuromuscular Control:**   - Proprioception refers to the body’s ability to sense joint position and movement. Enhancing this can help prevent dislocations or subluxations. - **Balance training** and **closed-chain exercises** (e.g., push-ups on unstable surfaces, stability ball exercises) help improve shoulder joint awareness and neuromuscular control, reducing the risk of instability.   **4. Improving Flexibility and Range of Motion:**   - Maintaining **flexibility** in the shoulder joint and surrounding muscles helps prevent abnormal movement patterns that can lead to instability. - Stretching exercises, such as **posterior shoulder stretches** and **cross-body stretches**, can help maintain flexibility and avoid excessive tightness that might contribute to instability.   **5. Using Proper Sports Technique:**   - Incorrect techniques in sports, especially in activities like throwing, swimming, or contact sports, can strain the shoulder joint. - Proper biomechanics, particularly for athletes involved in overhead or contact sports (e.g., baseball, tennis, football), help prevent undue stress on the shoulder. Working with a coach or trainer to perfect technique can be key in injury prevention.   **6. Avoiding Overhead Activity Overuse:**   - Repetitive overhead movements can strain the shoulder, especially in sports like swimming, tennis, or baseball. - Implementing **rest periods** and limiting excessive repetition of overhead motions can reduce the risk of shoulder strain and instability.   **7. Wearing Protective Gear (if applicable):**   - Athletes in contact sports (e.g., football, rugby) may benefit from wearing **shoulder braces or supports** to help stabilize the shoulder during competition and reduce the risk of injury.   **8. Proper Warm-Up and Cool-Down:**   - Warming up before exercise increases blood flow to muscles and prepares the joints for activity, reducing the risk of injury. - A good warm-up routine should include dynamic stretching and shoulder mobility exercises. - A **cool-down** with stretching helps maintain flexibility and recover muscle function, reducing tightness and post-activity strain.   **9. Rehabilitation After Injury:**   - If you've had a previous shoulder injury, working with a physical therapist to properly rehabilitate the shoulder is crucial. - Gradual return to activity with a focus on strengthening, flexibility, and stability exercises helps prevent future instability events.   **10. Avoid High-Risk Positions:**   - Limiting movements that put the shoulder in a vulnerable position (e.g., extreme abduction and external rotation) can reduce the risk of anterior shoulder instability, especially for those prone to dislocations.   By focusing on shoulder stability, strength, and technique, many individuals can prevent or reduce the risk of developing anterior shoulder instability. | Preventing anterior shoulder instability, particularly in individuals who are at risk due to sports, activity levels, or previous injuries, involves strengthening the muscles around the shoulder joint, improving flexibility, and practicing proper movement mechanics. Here are key strategies for prevention:  **1. Strengthening the Shoulder Musculature**   - **Rotator Cuff Exercises**: Strengthening the muscles of the rotator cuff (supraspinatus, infraspinatus, teres minor, subscapularis) helps stabilize the shoulder joint.   - *Exercises*: External and internal rotation with resistance bands or weights, dumbbell shoulder raises, and cable exercises. - **Scapular Stabilization**: Strengthening the muscles around the scapula (shoulder blade) provides a solid base for shoulder movements.   - *Exercises*: Scapular retraction (pinching shoulder blades together), rows, and serratus anterior exercises like wall slides.   **2. Improving Shoulder Flexibility and Range of Motion**   - **Stretching**: Tightness in the shoulder muscles can increase the risk of instability. Stretching the chest, shoulder, and upper back muscles can help maintain balanced flexibility.   - *Examples*: Sleeper stretch, cross-body shoulder stretch, and doorway pec stretch. - **Joint Mobilization**: Exercises that promote smooth movement of the shoulder joint through its full range of motion can help prevent stiffness that may contribute to instability.   **3. Proper Technique and Biomechanics**   - **Sport-Specific Technique**: Learning proper movement mechanics for sports or activities that involve repetitive overhead movements (like baseball, tennis, or swimming) can reduce the risk of shoulder instability. - **Core and Postural Training**: Core stability and good posture also contribute to proper shoulder function. Exercises like planks, side planks, and posture-focused routines help maintain shoulder alignment.   **4. Progressive Loading and Recovery**   - **Gradual Progression**: Avoid overloading the shoulder with excessive weight or force without proper conditioning. Increase intensity gradually in strength training or sports activities to allow the shoulder to adapt. - **Adequate Rest**: Allow enough recovery time between activities to prevent overuse injuries, which can weaken the shoulder muscles and ligaments, leading to instability.   **5. Taping or Bracing (in high-risk situations)**   - **Shoulder Bracing**: In athletes or individuals with a history of shoulder instability, using a brace or taping during high-risk activities can provide additional external support and prevent dislocations.   **6. Addressing Underlying Factors**   - **Post-Injury Rehabilitation**: If a person has experienced shoulder instability or dislocation before, adhering to a structured rehabilitation program is crucial for full recovery and prevention of re-injury. This may involve physical therapy to address any muscle imbalances or joint laxity. - **Regular Check-Ups with a Specialist**: For individuals involved in high-risk activities, regular evaluations with an orthopedic specialist or physical therapist can help identify any signs of instability before they worsen.   By focusing on these areas, you can strengthen the shoulder and reduce the risk of anterior shoulder instability, which is especially important for athletes and individuals with previous shoulder injuries. | This is for informational purposes only. For medical advice or diagnosis, consult a professional.  Here are some ways to prevent anterior shoulder instability:   - **Strengthen the muscles around your shoulder joint.** This includes the rotator cuff muscles, as well as the muscles that stabilize your shoulder blade. Strong muscles can help to keep your shoulder joint in place.  ([ref](https://orthoinfo.aaos.org/en/recovery/rotator-cuff-and-shoulder-conditioning-program/#:~:text=Strength%3A%20Strengthening%20the%20muscles%20that,keep%20your%20shoulder%20joint%20stable.)) - **Avoid activities that put your shoulder at risk for injury.(**[**ref**](https://www.leedsth.nhs.uk/patients/resources/anterior-shoulder-dislocation-without-bony-injury/#:~:text=In%20the%20early%20days%20you,high%20five'%20position%2C%20putting%20your)**)** This may include contact sports, such as football and hockey, as well as activities that involve repetitive overhead motions, such as baseball pitching and volleyball serving. - **Warm up before participating in any physical activity.** This will help to loosen your muscles and joints, and make them less susceptible to injury. ([ref](https://www.heart.org/en/healthy-living/fitness/fitness-basics/warm-up-cool-down#:~:text=It%20increases%20the%20temperature%20and,and%20safer%20during%20your%20workout.)) - **Use proper technique when participating in sports or other activities.** This will help to reduce your risk of injury. - **If you have had a shoulder injury in the past, follow your doctor's instructions carefully.** This may include wearing a sling or brace, and avoiding certain activities.   Here are some specific exercises that you can do to strengthen the muscles around your shoulder joint:   - **Rotator cuff exercises:** These exercises can be done with resistance bands or light weights. ([ref](https://www.verywellhealth.com/shoulder-rotator-cuff-strengthening-exercises-2696618#:~:text=One%20of%20the%20best%20ways,cuff%20exercises%20with%20resistance%20bands.)) They include internal rotation, external rotation, and abduction. ([ref](https://pubmed.ncbi.nlm.nih.gov/19769415/#:~:text=The%20rotator%20cuff%20performs%20multiple,and%20internal%20rotation%20(IR).)) - **Scapular stabilization exercises:** These exercises help to strengthen the muscles that stabilize your shoulder blade. They include push-ups, rows, and pull-ups. ([ref](https://www.cuh.nhs.uk/patient-information/scapular-stabilisation-exercises/))   If you have any concerns about shoulder instability, talk to your doctor. They can help you develop a prevention plan that is right for you. |
| **If I’ve dislocated my shoulder once, am I more likely to dislocate it again?** | Yes, if you have dislocated your shoulder once, you are more likely to dislocate it again. This increased risk of recurrent dislocation is due to several factors that are common after an initial dislocation:  **1. Damage to Stabilizing Structures:**   - A shoulder dislocation often results in damage to the soft tissues that stabilize the joint, such as the **labrum** (a rim of cartilage that deepens the shoulder socket) and the **capsule** (the fibrous tissue that surrounds the joint). - A **Bankart lesion**, which is a tear of the labrum, is a common injury following anterior shoulder dislocation and increases the likelihood of recurrent dislocations. - Ligaments may become stretched or torn, making it harder for the joint to remain stable during movement.   **2. Muscle Weakness and Imbalance:**   - After a dislocation, the muscles around the shoulder, particularly the **rotator cuff** and **scapular stabilizers**, may weaken due to injury or disuse during the recovery period. Without proper rehabilitation, this weakness can persist, leading to instability. - Imbalances in the shoulder muscles can make it harder for the joint to stay in its proper position, increasing the chance of future dislocations.   **3. Proprioception Impairment:**   - The ability of your body to sense the position of your shoulder joint (**proprioception**) can be affected after a dislocation. This makes it more difficult for the muscles to respond quickly and stabilize the joint during activity, increasing the risk of reinjury.   **4. Age and Activity Level:**   - **Younger individuals** (especially under 30) are at a higher risk of recurrent shoulder dislocations after the first event. This is because younger people tend to have more active lifestyles and may engage in activities that stress the shoulder joint. - Athletes, particularly those involved in contact sports or activities that involve overhead motion (e.g., football, basketball, swimming), are also at increased risk of re-dislocation.   **5. Joint Laxity or Hypermobility:**   - Some individuals have naturally loose or hypermobile joints, which can make the shoulder more prone to dislocation. If you have joint laxity, you may be at higher risk of recurrent dislocations.   **Preventing Recurrent Dislocation**  To reduce the risk of future shoulder dislocations, it's important to follow a structured rehabilitation program that focuses on:   - **Strengthening the rotator cuff** and scapular stabilizers. - **Improving shoulder stability** through exercises that enhance proprioception and neuromuscular control. - **Maintaining flexibility** in the shoulder joint without overstretching. - **Avoiding high-risk movements** or situations that put excessive strain on the shoulder, especially during recovery.   In some cases, recurrent shoulder dislocations may require surgical intervention, particularly if conservative measures like physical therapy are not effective. Surgery (e.g., arthroscopic Bankart repair) can help restore stability to the joint by repairing damaged ligaments and the labrum.  If you've dislocated your shoulder, it's crucial to work with a healthcare provider or physical therapist to reduce your chances of recurrence. | Yes, once you've dislocated your shoulder, you're more likely to dislocate it again. This is because the initial dislocation can stretch or tear the ligaments, muscles, or tendons that help stabilize the shoulder joint. After a dislocation, the joint may become less stable, increasing the risk of future dislocations, especially if proper rehabilitation and strengthening exercises aren’t done.  The likelihood of re-dislocation depends on several factors:   - **Age**: Younger individuals, especially those under 20, are more prone to repeated dislocations due to more elastic tissues. - **Activity level**: Participation in contact sports or activities that put stress on the shoulder increases the risk. - **Degree of injury**: The severity of the initial dislocation and whether there was significant damage to the soft tissues or bone also impacts the risk of recurrence.   To reduce the risk of future dislocations, it’s important to follow a tailored physical therapy program that focuses on strengthening the rotator cuff muscles and stabilizing the shoulder. In some cases, surgery may be necessary if the shoulder remains unstable.  Would you like guidance on shoulder-strengthening exercises or to explore options for seeing an orthopedic specialist? | This is for informational purposes only. For medical advice or diagnosis, consult a professional.  Yes, you are more likely to dislocate your shoulder again if you have dislocated it once before. This is because the muscles and soft tissues that hold your shoulder joint in place can become stretched or torn during the first dislocation. This makes the joint weaker and more prone to further dislocations.  The risk of re-dislocation is higher in young people and athletes who participate in contact sports. If you have dislocated your shoulder once, it is important to take steps to prevent it from happening again. These steps may include: ([ref](https://www.ncbi.nlm.nih.gov/books/NBK459125/))   - Wearing a sling or brace to support your shoulder for a few weeks after the injury.  ([ref](https://www.hopkinsmedicine.org/health/conditions-and-diseases/shoulder-instability#:~:text=If%20the%20pain%20is%20significant,shoulder%20bracing%20may%20also%20be)) - Doing physical therapy exercises to strengthen the muscles around your shoulder.  ([ref](https://www.uhdb.nhs.uk/shoulder-instabilitydislocation/)) - Avoiding activities that put your shoulder at risk of injury, such as contact sports.   If you have dislocated your shoulder more than once, you may need surgery to repair the damaged tissues and stabilize the joint. ([ref](https://www.mayoclinic.org/diseases-conditions/dislocated-shoulder/diagnosis-treatment/drc-20371720#:~:text=Surgery.,dislocations%20despite%20strengthening%20and%20rehabilitation.)) |
| **What are the symptoms of anterior shoulder instability?** | Anterior shoulder instability occurs when the head of the humerus (upper arm bone) slips out of the front of the shoulder joint, often due to trauma or repetitive overuse. Common symptoms of anterior shoulder instability include:   1. **Pain**:    - Especially during or after shoulder movement, particularly with overhead activities.    - Sudden pain during dislocation or subluxation (partial dislocation). 2. **Sensation of Shoulder "Slipping" or "Giving Out"**:    - A feeling that the shoulder might pop out of place, especially when the arm is in a vulnerable position, like being raised overhead or rotated outward. 3. **Limited Range of Motion**:    - Difficulty lifting the arm or performing movements that involve external rotation or abduction (moving the arm away from the body). 4. **Weakness**:    - A sense of weakness or lack of strength in the shoulder, especially when performing pushing or overhead activities. 5. **Recurrent Dislocations or Subluxations**:    - A history of the shoulder repeatedly dislocating or partially dislocating, especially with physical activities or certain movements. 6. **Swelling or Bruising** (in acute cases):    - Following an injury, the shoulder may appear swollen or bruised. 7. **Apprehension Sign**:    - Fear or discomfort when the shoulder is moved into certain positions, particularly external rotation and abduction, as this may cause a sensation that the shoulder will dislocate.   If not managed properly, recurrent anterior shoulder instability can lead to chronic pain, weakness, and long-term shoulder dysfunction. | Anterior shoulder instability occurs when the shoulder joint is prone to slipping or dislocating due to looseness in the structures that stabilize the joint, particularly at the front (anterior) of the shoulder. Common **symptoms** include:   1. **Pain**: Especially in the front of the shoulder, often aggravated by overhead or throwing activities. 2. **Recurrent Dislocations**: A feeling that the shoulder slips out of place or dislocates, particularly when the arm is positioned in abduction and external rotation (like throwing a ball). 3. **Apprehension**: A sensation of instability or fear that the shoulder will pop out, especially in certain positions (e.g., arm raised and rotated outward). 4. **Weakness**: Difficulty in maintaining strength in certain shoulder movements, particularly overhead or rotational activities. 5. **Clicking or Popping**: Some people experience clicking, catching, or popping sensations with shoulder movement. 6. **Decreased Range of Motion**: Limitation in the full range of shoulder movement, particularly when trying to rotate the arm outwards. 7. **Muscle Guarding**: Surrounding muscles may tighten up to compensate for instability.   If you or someone is experiencing these symptoms, it is important to get evaluated by an orthopedic specialist for proper diagnosis and treatment. | This is for informational purposes only. For medical advice or diagnosis, consult a professional.  Anterior shoulder instability is a condition where the shoulder joint, specifically the ball (humeral head) and socket (glenoid), becomes loose and prone to dislocation.  ([ref](https://www.hopkinsmedicine.org/health/conditions-and-diseases/shoulder-instability#:~:text=People%20with%20instability%20of%20the,This%20is%20commonly%20associated%20with))  **Common Symptoms:**   - **Pain:** This can range from a dull ache to sharp, intense pain, especially during certain movements or activities.  ([ref](https://www.massgeneral.org/orthopaedics/sports-medicine/conditions-and-treatments/shoulder-instability)) - **Instability:** A feeling that the shoulder is slipping out of place or "giving way."  ([ref](https://www.hopkinsmedicine.org/health/conditions-and-diseases/shoulder-instability#:~:text=People%20with%20instability%20of%20the,This%20is%20commonly%20associated%20with)) - **Popping or clicking:** You might hear or feel these sensations in the shoulder joint. - **Weakness:** Difficulty lifting, reaching, or rotating the arm. - **Limited range of motion:** Stiffness or difficulty moving the shoulder through its full range.   ([ref](https://www.hopkinsmedicine.org/health/conditions-and-diseases/shoulder-instability#:~:text=Additional%20symptoms%20can%20include%20a,shoulder%20motion%2C%20swelling%20and%20bruising.)) - **Numbness or tingling:** This can occur if nerves are affected.   ([ref](https://lonestarneurology.net/neurological-disorders/burning-and-tingling-in-shoulders/#:~:text=Usually%2C%20it%20has%20a%20feeling,blood%20vessels%20in%20the%20area.))   **Causes:**   - **Traumatic injury:** A fall or blow to the shoulder can cause the ball to pop out of the socket, stretching or tearing ligaments and tendons. ([ref](https://www.mayoclinic.org/diseases-conditions/dislocated-shoulder/symptoms-causes/syc-20371715#:~:text=The%20ligaments%20%E2%80%94%20tissue%20that%20joins,often%20making%20the%20dislocation%20worse.)) - **Repetitive overuse:** Overhand motions in sports like baseball, volleyball, or swimming can loosen the shoulder joint over time.  ([ref](https://orthoinfo.aaos.org/en/diseases--conditions/shoulder-injuries-in-the-throwing-athlete/)) - **Loose ligaments:** Some people have naturally looser ligaments, making them more prone to shoulder instability. ([ref](https://www.hopkinsmedicine.org/health/conditions-and-diseases/shoulder-instability))   **Diagnosis:**  A doctor will examine your shoulder, checking for range of motion, strength, and signs of instability. ([ref](https://www.hopkinsmedicine.org/health/conditions-and-diseases/shoulder-instability#:~:text=Diagnosing%20shoulder%20instability%20includes%20a,out%20other%20causes%20of%20shoulder)) They may also order imaging tests like X-rays or MRIs to confirm the diagnosis and rule out other injuries.  **Treatment:**  Treatment depends on the severity of the instability. Options include:   - **Physical therapy:** Exercises to strengthen the muscles around the shoulder and improve stability.   ([ref](https://orthoinfo.aaos.org/en/recovery/rotator-cuff-and-shoulder-conditioning-program/#:~:text=Strength%3A%20Strengthening%20the%20muscles%20that,keep%20your%20shoulder%20joint%20stable.)) - **Medications:** Pain relievers and anti-inflammatory drugs to reduce pain and swelling. ([ref](https://www.hopkinsmedicine.org/health/conditions-and-diseases/shoulder-instability#:~:text=NSAIDs%20(nonsteroidal%20anti%2Dinflammatory%20drugs,Aleve%20can%20be%20used%20to)) - **Surgery:** In some cases, surgery may be needed to repair torn ligaments or tighten the shoulder joint. ([ref](https://www.uhs.nhs.uk/departments/trauma-and-orthopaedics/shoulders/patient-information/anterior-stabilisation-of-the-shoulder#:~:text=This%20involves%20repair%20of%20the,over%2Dstretched%20and%20damaged%20ligaments.))   **If you think you might have anterior shoulder instability, see a doctor for diagnosis and treatment.** |
| **How is anterior shoulder instability diagnosed?** | Anterior shoulder instability is diagnosed through a combination of patient history, physical examination, and imaging studies. The process typically involves the following steps:  **1. Patient History**   - **Symptoms**: The patient may describe a sensation of the shoulder "slipping" or "popping out" of place, often during specific activities, such as overhead movements or throwing. Pain, weakness, and a feeling of instability or looseness in the shoulder are common. - **Injury Mechanism**: The physician will ask about any history of trauma, such as a fall or direct blow to the shoulder, or repetitive motions that may have contributed to the instability. - **Previous Dislocations**: A history of previous shoulder dislocations or subluxations (partial dislocations) is important. Patients may describe situations where the shoulder spontaneously relocated or required medical assistance for reduction.   **2. Physical Examination**  Several clinical tests are used to assess anterior shoulder instability. These include:   - **Apprehension Test**: The patient’s arm is positioned in abduction and external rotation, a position where the shoulder is more vulnerable to dislocation. If the patient feels apprehension or fear that the shoulder might dislocate, it is a positive sign. - **Relocation Test**: After the apprehension test, if the examiner applies pressure to the anterior part of the shoulder, and this relieves the patient's fear or discomfort, it indicates anterior instability. - **Anterior Drawer Test**: The physician applies an anterior force to the humeral head to check for increased movement or instability of the shoulder joint. - **Sulcus Sign**: This test involves pulling the arm downward while the patient is relaxed. If a noticeable "sulcus" or gap appears below the acromion, it indicates instability. - **Load and Shift Test**: The physician applies a load to the humeral head and attempts to shift it anteriorly. Increased mobility or abnormal movement suggests instability.   **3. Imaging Studies**  Imaging is used to assess the structures of the shoulder and confirm the diagnosis.   - **X-rays**: These are typically the first imaging test ordered to check for bone abnormalities, such as fractures or bone loss (especially of the glenoid or humeral head). X-rays can also show any previous dislocations. - **MRI (Magnetic Resonance Imaging)**: This is used to evaluate soft tissue structures like the labrum, ligaments, and rotator cuff muscles. An MRI may show a **Bankart lesion** (a tear of the labrum from the glenoid) or a **Hill-Sachs lesion** (a dent in the humeral head caused by dislocation). - **CT Scan**: In some cases, a CT scan may be performed to better visualize bone structure or assess bone loss on the glenoid or humerus. - **MRI Arthrogram**: Sometimes, a contrast dye is injected into the shoulder joint before MRI to better highlight injuries to the labrum or ligaments.   **4. Specialized Tests**   - **Electromyography (EMG)**: In cases where nerve injury is suspected due to the shoulder instability, an EMG may be ordered to evaluate the function of the nerves supplying the shoulder muscles.   **5. Severity and Chronicity Assessment**   - The physician will also assess whether the instability is acute (due to a recent injury) or chronic (recurrent episodes of dislocation or subluxation). This can influence both diagnosis and treatment.   In summary, anterior shoulder instability is diagnosed through careful evaluation of symptoms, clinical tests to assess joint stability, and imaging to confirm the presence of structural injuries. | Anterior shoulder instability is diagnosed through a combination of **patient history**, **physical examination**, and **imaging studies**. Here’s a breakdown of the process:  **1. Patient History**  The doctor will ask about:   - History of trauma or injury (such as dislocation). - Recurrent shoulder dislocations or subluxations (partial dislocations). - Feeling of looseness or instability in the shoulder. - Any associated pain, especially during activities that involve overhead motion or external rotation. - Activities or sports that could have caused excessive strain on the shoulder.   **2. Physical Examination**  The physician performs specific tests to assess shoulder stability, including:   - **Apprehension Test**: The arm is placed in an abducted and externally rotated position, which may reproduce the sensation of instability or dislocation, causing apprehension in the patient. - **Relocation Test**: While in the apprehension position, a posteriorly directed force on the shoulder is applied. Relief of the patient’s apprehension or pain indicates anterior instability. - **Anterior Drawer Test**: The physician moves the humeral head anteriorly to see if it translates excessively, indicating laxity or instability. - **Sulcus Sign**: The doctor pulls down on the arm while it's in a relaxed position, checking for a visible or palpable indentation under the acromion, which may indicate shoulder laxity.   **3. Imaging Studies**   - **X-rays**: To assess for any bone abnormalities, fractures, or damage related to shoulder dislocations. - **MRI (Magnetic Resonance Imaging)**: Helps in detecting soft tissue injuries, such as labral tears (e.g., a **Bankart lesion**, common in anterior instability) or ligamentous damage. - **CT Scan**: Provides detailed images of the bony anatomy, helping identify any structural damage to the glenoid or humeral head. - **MR Arthrogram**: A contrast dye is injected into the joint to better visualize labral tears and other soft tissue abnormalities.   **4. Special Tests**   - **Electromyography (EMG)** may be used if there’s suspicion of nerve involvement or muscle weakness associated with the shoulder instability.   A combination of these diagnostic methods helps determine the severity and cause of the anterior shoulder instability, guiding the treatment plan. | This is for informational purposes only. For medical advice or diagnosis, consult a professional.  Anterior shoulder instability is diagnosed through a combination of methods:   - **Medical History:** The doctor will ask about any previous shoulder injuries, dislocations, or subluxations, as well as any other relevant medical conditions. ([ref](https://www.massgeneral.org/orthopaedics/sports-medicine/conditions-and-treatments/shoulder-instability#:~:text=Shoulder%20Instability%20Diagnosis&They%20will%20also%20ask%20you%20about%20injuries%20and%20shoulder%20instability%20history.)) - **Physical Examination:** The doctor will assess the range of motion, strength, and stability of the shoulder joint. ([ref](https://www.hopkinsmedicine.org/health/conditions-and-diseases/shoulder-instability)) They may perform specific tests to check for laxity or apprehension in the shoulder. ([ref](https://www.londonarmdoc.com/anterior-shoulder-instability-shoulder-surgeon-london/)) - **Imaging Tests:** X-rays, CT scans, or MRIs may be used to visualize the bones and soft tissues of the shoulder joint and identify any damage or abnormalities.   ([ref](https://www.radiologyinfo.org/en/info/shouldermr#:~:text=MRI%20of%20the%20shoulder%20provides,and%20vessels%2C%20from%20any%20angle.)) ([ref](https://pmc.ncbi.nlm.nih.gov/articles/PMC5594016/#:~:text=CT%20arthrography%20has%20also%20been,injuries%20related%20to%20anterior%20shoulder))   If you are experiencing symptoms of anterior shoulder instability, it is important to see a doctor for an accurate diagnosis and treatment plan. |
| **What are the potential long-term effects of anterior shoulder instability?** | Anterior shoulder instability occurs when the head of the humerus (upper arm bone) slips out of the front of the shoulder socket, typically due to trauma or repetitive overuse. If not treated properly, it can lead to several potential long-term effects, including:  Chronic Shoulder Instability: Repeated dislocations or subluxations (partial dislocations) can cause ongoing instability, making the shoulder more prone to dislocation even during minor activities. This can reduce the function and stability of the shoulder over time.  Joint Damage and Arthritis: Repeated dislocations can damage the cartilage and soft tissues within the shoulder joint, leading to degenerative changes such as osteoarthritis. This can cause pain, stiffness, and reduced range of motion in the shoulder.  Labral Tears (Bankart Lesion): The labrum is a ring of cartilage that surrounds the shoulder socket, providing stability. Anterior dislocations can cause a Bankart lesion, where the labrum is torn. This can impair shoulder function and increase the risk of recurrent dislocations.  Muscle Weakness and Atrophy: Ongoing instability may lead to weakness in the muscles around the shoulder, particularly the rotator cuff and the deltoid muscle. Long-term disuse or overcompensation by other muscles may result in muscle atrophy, leading to decreased shoulder strength and function.  Decreased Range of Motion: Over time, repeated dislocations or subluxations can cause the shoulder's range of motion to become restricted, which can affect daily activities and sports performance.  Nerve Damage: In some cases, shoulder dislocations can cause damage to the nerves around the shoulder, such as the axillary nerve. This can result in numbness, tingling, or weakness in the arm and shoulder.  Impaired Quality of Life: Chronic shoulder instability can limit activities, both in sports and daily tasks, which can reduce the overall quality of life. Fear of dislocation may also lead to avoidance of certain movements or activities.  Long-term management of anterior shoulder instability often requires physical therapy to strengthen the shoulder muscles, and in some cases, surgery to stabilize the joint and repair damaged structures like the labrum. Early and appropriate treatment is key to preventing these long-term effects. | Anterior shoulder instability occurs when the head of the humerus (upper arm bone) repeatedly slips out of its socket in the shoulder joint. It is often caused by a traumatic injury like a shoulder dislocation, but can also result from overuse in sports involving repetitive overhead movements.  The potential long-term effects of untreated or poorly managed anterior shoulder instability include:   1. **Recurrent Dislocations**: Once the shoulder has dislocated, there is an increased risk of repeated dislocations, especially with physical activities or even with minor movements. 2. **Glenoid Labrum Damage (Bankart Lesion)**: A tear in the labrum, the cartilage around the edge of the shoulder socket, can occur with initial dislocations. Recurrent dislocations can worsen this damage, making the shoulder less stable. 3. **Hill-Sachs Lesion**: This is a bony injury to the humeral head that occurs when it impacts the edge of the shoulder socket during a dislocation. It can further compromise the stability of the shoulder. 4. **Rotator Cuff Tears**: Repeated shoulder instability increases the likelihood of damage to the rotator cuff tendons, particularly in older individuals. 5. **Shoulder Osteoarthritis**: Chronic instability can lead to accelerated wear of the cartilage in the joint, increasing the risk of early-onset osteoarthritis. This causes pain, stiffness, and decreased range of motion over time. 6. **Weakness and Loss of Function**: Recurrent instability can lead to muscle atrophy and weakness, particularly in the muscles surrounding the shoulder, like the rotator cuff and deltoid. This can limit arm strength and mobility. 7. **Chronic Pain**: Ongoing instability can lead to inflammation of the shoulder structures, causing persistent pain, even when the shoulder is not actively dislocating. 8. **Decreased Quality of Life**: The inability to perform normal activities, particularly in athletes or individuals with physically demanding jobs, can lead to significant functional limitations and emotional distress.   Management strategies typically include physical therapy to strengthen the shoulder muscles, lifestyle modifications, and in severe cases, surgical interventions like labral repair or shoulder stabilization surgery to prevent further damage. | This is for informational purposes only. For medical advice or diagnosis, consult a professional.  Anterior shoulder instability, if left untreated, can lead to a number of long-term problems. ([ref](https://www.ncbi.nlm.nih.gov/books/NBK538234/)) The most common is recurrent shoulder dislocations. This can happen when the ligaments or labrum (a ring of cartilage that helps to stabilize the shoulder joint) are stretched or torn. ([ref](https://www.hopkinsmedicine.org/health/conditions-and-diseases/shoulder-instability#:~:text=The%20capsule%2C%20ligaments%20or%20labrum,during%20shoulder%20subluxation%20and%20dislocation.)) Once the shoulder has dislocated once, it is more likely to dislocate again. ([ref](https://www.nhs.uk/conditions/dislocated-shoulder/#:~:text=Once%20you%20dislocate%20your%20shoulder,well%20your%20shoulder%20joint%20healed.))  Other long-term effects of anterior shoulder instability can include:   - **Shoulder pain:** This can be a dull ache or a sharp pain that occurs with certain movements.  ([ref](https://www.mayoclinic.org/symptom-checker/shoulder-pain-in-adults-adult/related-factors/itt-20009075)) - **Shoulder weakness:** This can make it difficult to lift or rotate the arm.   ([ref](https://www.nhslanarkshire.scot.nhs.uk/services/physiotherapy-msk/shoulder-instability/#:~:text=There%20is%20a%20chance%20your,stiffness%2C%20giving%20way%20and%20pain.)) - **Shoulder stiffness:** This can make it difficult to move the arm through its full range of motion.   ([ref](https://pmc.ncbi.nlm.nih.gov/articles/PMC7075754/#:~:text=Less%20activity%2C%20loss%20of%20capsule,after%20traumatic%20anterior%20shoulder%20instability.)) - **Arthritis:** This is a condition that causes inflammation and pain in the joints. ([ref](https://www.nhs.uk/conditions/arthritis/#:~:text=Arthritis%20is%20a%20common%20condition,and%20inflammation%20in%20a%20joint.)) Anterior shoulder instability can increase the risk of developing arthritis in the shoulder joint.  ([ref](https://pmc.ncbi.nlm.nih.gov/articles/PMC8999818/#:~:text=Anterior%20subluxation%20after%20reduction%20might,contraindication%20for%20nonoperative%20treatment%20%5B23%2C)) - **Nerve damage:** This cancause numbness, tingling, or weakness in the arm or hand.  ([ref](https://www.uofmhealth.org/conditions-treatments/cmc/hand-elbow-wrist/nerve-injuries-hand-wrist-and-elbow)) - **Blood vessel damage:** This can lead to bleeding or blood clots.   The best way to prevent long-term problems from anterior shoulder instability is to get treatment as soon as possible after the initial injury. Treatment may include physical therapy, surgery, or both. ([ref](https://www.hopkinsmedicine.org/health/conditions-and-diseases/shoulder-instability#:~:text=Treatment%20for%20shoulder%20instability%20includes%20both%20non%2Doperative%20and%20surgical%20options.)). |
| **When is surgery necessary for anterior shoulder instability?** | Surgery for anterior shoulder instability may become necessary when conservative treatments, such as physical therapy, rest, and lifestyle modifications, fail to provide relief or when the risk of recurrent dislocations is high. Specific situations in which surgery may be indicated include:  Recurrent Shoulder Dislocations: If the shoulder dislocates multiple times, particularly after conservative treatments, surgery is often recommended to stabilize the joint and prevent further dislocations.  Significant Structural Damage: If there is a substantial injury to the bones, ligaments, or soft tissue structures that stabilize the shoulder (such as a Bankart lesion, Hill-Sachs lesion, or glenoid bone loss), surgery may be required to repair or reconstruct these structures.  Failed Non-Surgical Treatment: If the patient continues to experience instability, pain, or limited function despite extensive physical therapy or other non-surgical measures, surgery may be considered.  Young, Active Individuals: Athletes or people involved in physically demanding activities, particularly those younger than 30 years, often have a higher risk of recurrent dislocation. In such cases, surgery is recommended earlier to prevent further damage and allow a return to activity.  High-Risk Sports or Occupations: Individuals engaged in contact sports (e.g., rugby, football) or physically demanding jobs (e.g., manual labor) may require surgery sooner to reduce the risk of injury recurrence during these activities.  The decision for surgery is individualized, depending on the patient's activity level, the extent of the damage, and the likelihood of recurrent instability. Common surgical options include arthroscopic Bankart repair, Latarjet procedure, or capsular shift surgery. | Surgery for **anterior shoulder instability** is typically considered necessary when non-surgical treatments fail or certain conditions are present. Here are the key scenarios in which surgery is usually recommended:  **1. Recurrent Dislocations**   - If you’ve had **multiple episodes** of shoulder dislocation, especially with minimal trauma or activity, surgery may be necessary to prevent further damage and restore stability.   **2. Significant Damage to Shoulder Structures**   - **Labral tears** (e.g., Bankart lesion) or **bone loss** (e.g., from the glenoid or humeral head) may require surgical repair, particularly if these injuries are contributing to instability. - Imaging (e.g., MRI, CT scans) can help identify this damage.   **3. Failure of Non-Surgical Treatment**   - If a trial of **conservative treatment** (like physical therapy, bracing, and activity modification) doesn’t result in stability or function improvement, surgery might be the next step. - Typically, 3 to 6 months of non-operative treatment is attempted before surgery is considered unless other factors suggest early intervention.   **4. High-Risk Individuals**   - People involved in **contact sports** (e.g., rugby, football) or professions requiring shoulder stability (e.g., manual laborers) are at a higher risk of recurrent dislocation and may be candidates for surgery earlier to avoid recurrent injury. - **Young patients** (especially those under 25) are also at higher risk of recurrence after initial dislocation, making surgical stabilization a stronger consideration.   **5. Severe Instability or Functional Impairment**   - If your shoulder instability is causing **significant limitations** in daily life, work, or sports and is not improving with therapy, surgery might help restore normal function and prevent further damage.   **6. Acute Traumatic Dislocations**   - In cases of a **first-time traumatic dislocation** accompanied by large tears or significant fractures (especially in younger, active individuals), early surgery may be advised to stabilize the shoulder and prevent recurrence.   **Surgical Options:**   - **Arthroscopic Bankart Repair**: To repair the labrum and associated structures. - **Latarjet Procedure**: For cases with bone loss, involving a bone graft from the shoulder blade to increase stability. - **Capsular Shift or Plication**: Tightens loose shoulder capsule tissue.   Ultimately, the decision for surgery depends on individual factors, including the severity of instability, physical demands, and response to non-surgical treatments. Consulting with an orthopedic specialist who can assess your specific condition through physical exams and imaging is critical for making the right choice. | This is for informational purposes only. For medical advice or diagnosis, consult a professional.  Surgery is typically recommended for anterior shoulder instability when non-surgical treatments, such as physical therapy and activity modification, have failed to provide adequate relief or when the instability is severe or recurrent.  Here are some specific situations where surgery may be considered:   - **Recurrent dislocations:** If the shoulder dislocates multiple times, surgery may be necessary to stabilize the joint and prevent future dislocations. ([ref](https://www.nhs.uk/conditions/dislocated-shoulder/#:~:text=Treatment%20for%20a%20dislocated%20shoulder&Sometimes%20you%20may%20also%20need,dislocating%20the%20same%20shoulder%20again.)) - **Significant labral tears:** The labrum is a ring of cartilage that helps to stabilize the shoulder joint. If the labrum is torn, surgery may be needed to repair it.  ([ref](https://www.hopkinsmedicine.org/health/conditions-and-diseases/shoulder-labrum-tear#:~:text=The%20labrum%20is%20a%20thick,deepens%20the%20socket%20and%20helps&text=Tears%20that%20are%20due%20to,the%20rim%20of%20the%20socket.)) - **Bone loss:** In some cases, repeated dislocations can lead to bone loss in the shoulder joint. Surgery may be needed to reconstruct the bone and restore stability to the joint. ([ref](https://pubmed.ncbi.nlm.nih.gov/37643239/#:~:text=The%20ROC%20curve%20analysis%20demonstrated,subluxation%20(Youden%20index%20%3D%200.59%2C)) - **High-demand activities:** Athletes who participate in overhead or contact sports may be at higher risk for recurrent dislocations. Surgery may be considered to stabilize the shoulder and allow them to return to their sport.  ([ref](https://pubmed.ncbi.nlm.nih.gov/38296450/#:~:text=Abstract,higher%20risk%20of%20shoulder%20dislocations.))   The type of surgery that is recommended will depend on the specific cause of the instability and the severity of the injury. Some common surgical procedures for anterior shoulder instability include:   - **Arthroscopic Bankart repair:** This minimally invasive procedure is used to repair the labrum and tighten the shoulder capsule.  ([ref](https://www.dr-meyer-orthopaedics.com/operations/shoulder/unstable-shoulder/arthorscopic-capsulolabral-repair/#:~:text=The%20procedure%20is%20performed%20arthroscopically,2%20to%203%20small%20incisions)) - **Latarjet procedure:** This open surgery involves transferring a piece of bone from the coracoid process to the glenoid to help stabilize the joint. ([ref](https://www.medstarhealth.org/services/coracoid-transfer-latarjet-for-shoulder-instability#:~:text=In%20this%20open%20surgery%20technique,your%20shoulder%20blade%20to%20the)) - **Remplissage procedure:** This procedure is used to fill in a Hill-Sachs lesion, which is a defect in the humeral head that can occur after a dislocation.   It is important to discuss the risks and benefits of surgery with your doctor to determine if it is the right treatment option for you. |
| **What are the non-surgical treatment options for anterior shoulder instability?** | Non-surgical treatment options for anterior shoulder instability primarily focus on rehabilitation, pain management, and strengthening the muscles surrounding the shoulder joint to improve stability. These treatments are generally recommended for individuals who have experienced mild shoulder instability or dislocations and are not candidates for surgery or wish to avoid surgery. The main non-surgical treatment approaches include:  **1. Physical Therapy**   - **Strengthening Exercises**: Target the rotator cuff and scapular stabilizing muscles to help provide better control and support for the shoulder joint.   - **Rotator Cuff Strengthening**: These exercises focus on muscles like the supraspinatus, infraspinatus, teres minor, and subscapularis, which help stabilize the head of the humerus in the glenoid cavity.   - **Scapular Stabilization**: Exercises for muscles like the serratus anterior and trapezius help improve posture and mechanics of the shoulder joint, reducing the risk of instability. - **Range of Motion (ROM) Exercises**: Gentle, progressive exercises to maintain and improve shoulder mobility while avoiding positions that provoke instability (e.g., excessive abduction and external rotation). - **Proprioception Training**: Exercises to improve the awareness of joint position and movement, which can help prevent future dislocations by enhancing reflexive muscle control.   **2. Activity Modification**   - Avoiding activities that place excessive stress on the shoulder joint, particularly those that involve repetitive overhead movements, high-impact sports, or positions that increase the risk of dislocation (e.g., abduction and external rotation).   **3. Bracing or Taping**   - **Shoulder Brace**: Wearing a brace to provide external support, especially during sports or activities that may cause instability. - **Kinesiology Taping**: Some individuals find benefit in kinesiology taping, which can help provide additional proprioceptive feedback and support during activity.   **4. Anti-inflammatory Medications (NSAIDs)**   - Nonsteroidal anti-inflammatory drugs (NSAIDs), such as ibuprofen or naproxen, may be prescribed or taken over-the-counter to reduce pain and inflammation after an injury or in cases of recurrent instability.   **5. Corticosteroid Injections**   - In some cases, corticosteroid injections may be used to reduce inflammation and pain in the shoulder joint, although this is usually a temporary measure and may not directly address instability.   **6. Education and Lifestyle Changes**   - Education about the condition and how to avoid positions or activities that provoke instability is crucial. Patients may need to alter their athletic or daily routines to avoid further injury. - **Posture Correction**: Maintaining proper shoulder posture is essential, especially for individuals with instability, as poor posture can exacerbate symptoms.   **7. Heat and Ice Therapy**   - **Ice Therapy**: Applying ice immediately after an injury or dislocation can reduce pain and swelling. - **Heat Therapy**: Applying heat before physical therapy or stretching exercises can help relax muscles and improve flexibility.   **8. Neuromuscular Reeducation**   - Neuromuscular training aims to improve the coordinated activation of muscles around the shoulder joint to enhance stability. Techniques such as biofeedback or electrical stimulation may be used to reinforce proper muscle function.   Non-surgical treatment is typically recommended for individuals with mild to moderate anterior shoulder instability, especially for first-time dislocations or individuals who do not engage in high-risk sports. If symptoms persist or instability recurs, surgical options may be considered. | Non-surgical treatment options for anterior shoulder instability primarily aim to strengthen the shoulder muscles, improve joint stability, and reduce the risk of recurrent dislocation. Here are the common non-surgical approaches:  1. Physical Therapy (PT)  Strengthening exercises: The primary focus is on strengthening the rotator cuff and scapular stabilizers (like the deltoids, trapezius, and serratus anterior). This helps to enhance shoulder stability.  Range of motion exercises: Aiming to restore full motion without stressing the joint. This is important after any period of immobilization.  Proprioception training: Enhancing neuromuscular control of the shoulder by improving the body's ability to sense joint position, which helps prevent further injury.  Functional training: Exercises that simulate real-world activities, especially for athletes or those engaged in repetitive overhead activities.  2. Activity Modification  Avoiding or modifying high-risk activities that may lead to further dislocations, such as overhead throwing, heavy lifting, or contact sports, especially during early rehabilitation.  3. Bracing  Shoulder brace or sling: Using a brace to stabilize the shoulder during high-risk activities or post-injury to limit excessive motion and prevent dislocation.  Bracing can be temporary while muscles are strengthened through therapy.  4. Anti-inflammatory Medications  Non-steroidal anti-inflammatory drugs (NSAIDs): Medications such as ibuprofen or naproxen may help reduce pain and inflammation during the acute phase or after overuse.  5. Corticosteroid Injections  For some individuals with recurrent pain or inflammation, a corticosteroid injection may reduce inflammation in the joint. However, this is more for pain management and doesn't address instability directly.  6. Activity-specific Training  Athletes may work with sports-specific rehabilitation specialists to retrain proper throwing mechanics or other motions that may have contributed to the instability.  7. Cryotherapy (Cold Therapy)  Applying ice or cold therapy can help reduce swelling and inflammation, particularly in the early stages after a dislocation or injury.  8. Patient Education  Educating the patient about shoulder anatomy, mechanisms of injury, and appropriate postural adjustments. This can help prevent further episodes of instability.  Who Benefits from Non-surgical Treatment?  First-time dislocators: Especially younger patients or those who are less active and have not had multiple dislocations.  Older individuals: Elderly patients or those who are not involved in high-demand sports or overhead activities may benefit more from non-surgical approaches.  Athletes or active individuals: May begin with non-surgical treatments but will require careful monitoring of progress, particularly if their goal is to return to sports.  Duration and Outcome  Non-surgical treatments typically take several months of consistent rehabilitation. However, if recurrent dislocations or severe instability persist, surgical intervention may become necessary.  Would you like guidance on specific exercises or a detailed rehabilitation program tailored to your needs? | This is for informational purposes only. For medical advice or diagnosis, consult a professional.  Nonsurgical treatment is the first course of action for most patients with anterior shoulder instability. The goals of nonsurgical treatment are to reduce pain and inflammation, restore shoulder motion, and strengthen the muscles around the shoulder.  Here are some of the most common nonsurgical treatment options for anterior shoulder instability:   - **Rest:** Avoid activities that aggravate the shoulder and allow it to heal. ([ref](https://www.hopkinsmedicine.org/health/conditions-and-diseases/shoulder-instability)) - **Ice:** Apply ice to the shoulder for 20 minutes at a time, several times a day, to reduce pain and inflammation. ([ref](https://www.nhslanarkshire.scot.nhs.uk/services/physiotherapy-msk/shoulder-instability/)) - **Physical therapy:** A physical therapist can teach you exercises to improve shoulder motion and strength. ([ref](https://orthoinfo.aaos.org/en/recovery/rotator-cuff-and-shoulder-conditioning-program/)) - **Medications:** Over-the-counter pain relievers, such as ibuprofen or acetaminophen, can help reduce pain and inflammation. ([ref](https://www.hopkinsmedicine.org/health/conditions-and-diseases/shoulder-instability#:~:text=NSAIDs%20(nonsteroidal%20anti%2Dinflammatory%20drugs,Aleve%20can%20be%20used%20to)) In some cases, your doctor may prescribe stronger pain medication.   ([ref](https://staging.brainscape.com/flashcards/peripheral-nerve-conditions-13050329/packs/21307176)) - **Injections:** In some cases, your doctor may inject a corticosteroid medication into the shoulder joint to reduce inflammation. ([ref](https://my.clevelandclinic.org/health/treatments/cortisone-shots-steroid-injections))   Nonsurgical treatment is often successful in relieving pain and improving shoulder function. However, if your shoulder instability is severe or if you have repeated dislocations, you may need surgery. ([ref](https://www.dukehealth.org/treatments/orthopaedics/shoulder-dislocation-and-instability#:~:text=If%20nonsurgical%20treatments%20and%20physical,dislocations%20and%20return%20you%20to)). |
| **Does immobilization play a role in managing anterior shoulder instability? If so, for how long?** | Yes, immobilization plays a role in managing anterior shoulder instability, particularly after a shoulder dislocation or in cases of recurrent instability. Immobilization is typically recommended as part of the initial treatment to allow the soft tissues, such as ligaments and the joint capsule, to heal. The purpose of immobilization is to reduce pain, prevent further injury, and support healing in the early stages.  **Duration of Immobilization:**   1. **First-time shoulder dislocation**:    - Immobilization is commonly recommended for about **1 to 3 weeks**, depending on the severity of the injury, the patient's age, and their level of activity.    - The shoulder is often immobilized in a sling or brace, either in internal rotation (traditional position) or external rotation (which may provide better alignment for certain soft tissue healing).    - Some studies suggest that shorter periods of immobilization (around 1 week) followed by early rehabilitation may be just as effective, especially in younger and active patients. 2. **Recurrent instability**:    - For patients with chronic or recurrent instability, immobilization may still be used after an acute episode but often for shorter periods, typically **1 to 2 weeks**.    - After the initial immobilization period, physical therapy focusing on strengthening the rotator cuff muscles and stabilizers of the shoulder is critical.   **Key Considerations:**   - **Younger patients (under 30 years)** are more likely to experience recurrent instability after a dislocation, so their management often focuses on a balance between immobilization and early strengthening. - **Older patients** might require longer immobilization to allow proper healing and reduce the risk of complications. - **Surgical management**: In cases where conservative treatment (like immobilization) does not prevent recurrence, surgical stabilization may be considered. Immobilization is also used after surgery to protect the repair, generally for about **4 to 6 weeks** postoperatively, followed by a structured rehabilitation program.   In summary, immobilization does play a role in the acute management of anterior shoulder instability, typically for **1 to 3 weeks** depending on the severity, patient age, and activity level. However, early rehabilitation is crucial for restoring function and preventing recurrence. | Yes, immobilization does play a role in managing **anterior shoulder instability**, particularly in the acute phase following a dislocation or subluxation. The primary goals of immobilization are to allow the damaged tissues (ligaments, labrum, joint capsule) to heal, reduce inflammation, and prevent further damage.  **Duration of Immobilization:**  The typical duration of immobilization can vary depending on several factors, such as:   - **Age** (younger patients tend to have a higher risk of recurrent instability) - **Severity of the injury** (whether there was a full dislocation or partial subluxation) - **Rehabilitation goals** - **Surgical vs. nonsurgical approach**   For nonsurgical treatment of acute anterior shoulder instability:   - **1 to 3 weeks** of immobilization is generally recommended for most patients.   - Some clinicians opt for **shorter periods (7-10 days)** to avoid shoulder stiffness, particularly in older patients.   - In younger patients, particularly athletes, immobilization may be extended up to **3 weeks** to allow for better healing and stabilization of the soft tissue structures.   After immobilization, **early mobilization** with a guided rehabilitation program is crucial to regain range of motion, strength, and stability. Rehabilitation usually includes strengthening of the rotator cuff and scapular stabilizers to help prevent recurrent instability.  If surgery is performed (such as for labral repair), the period of immobilization might be longer, typically **4 to 6 weeks**, followed by a structured rehabilitation plan.  Would you like guidance on specific rehabilitation exercises following shoulder instability? | This is for informational purposes only. For medical advice or diagnosis, consult a professional.  Yes, immobilization plays a role in managing anterior shoulder instability. It is typically used after a shoulder dislocation to allow the injured tissues to heal and reduce the risk of re-injury.  The duration of immobilization varies depending on the severity of the injury and the individual's needs. In general, immobilization is recommended for 3-6 weeks. However, some studies have shown that shorter periods of immobilization (1-3 weeks) may be just as effective as longer periods.  The position of immobilization is also important. Traditionally, the arm has been immobilized in internal rotation. However, recent studies have shown that immobilization in external rotation may be more effective in reducing the risk of recurrence. ([ref](https://pubmed.ncbi.nlm.nih.gov/32596407/#:~:text=ER%20immobilization%20significantly%20reduced%20the,007).))  It is important to follow the advice of your doctor or physical therapist regarding the duration and position of immobilization. They will be able to recommend the best course of treatment for your individual needs. |
| **What are the surgical treatment options for anterior shoulder instability?** | Surgical treatment for anterior shoulder instability is typically considered when conservative treatments (such as physical therapy or activity modification) fail, or if the instability is recurrent and affects daily activities or sports performance. There are several surgical options, each with specific indications based on the severity of the instability, the patient’s activity level, and any associated damage (e.g., to the labrum, capsule, or bone). The main surgical options include:  **1. Arthroscopic Bankart Repair**   - **Indication**: This is the most common surgery for anterior shoulder instability, particularly in cases of a labral tear (Bankart lesion) from a dislocation. - **Procedure**: The labrum is reattached to the glenoid (socket) using sutures, often done arthroscopically (minimally invasive). This tightens the capsule and restores the stability of the shoulder. - **Recovery**: Generally faster than open surgery, but still requires several months of rehabilitation to regain full strength and range of motion.   **2. Latarjet Procedure**   - **Indication**: This is often indicated in patients with significant bone loss on the anterior glenoid rim (glenoid bone defects) or recurrent dislocations after a Bankart repair. It is also recommended in high-demand athletes. - **Procedure**: A piece of bone (coracoid process) along with its attached muscles and ligaments is transferred to the front of the glenoid to increase its size and improve stability. This provides both a bony block and muscle sling effect. - **Recovery**: The recovery may take longer than with arthroscopic Bankart repair, but it is highly effective in preventing further dislocations.   **3. Remplissage Procedure**   - **Indication**: This is used for patients with a large Hill-Sachs lesion (a bony defect on the humeral head caused by recurrent dislocations). It is often performed in combination with a Bankart repair. - **Procedure**: The infraspinatus tendon is transferred to fill the Hill-Sachs lesion, preventing the defect from engaging with the glenoid and causing dislocations. - **Recovery**: Similar to Bankart repair, with physical therapy focusing on shoulder stability and mobility.   **4. Open Bankart Repair**   - **Indication**: This is indicated for patients with extensive soft tissue or capsular damage or in cases where arthroscopic repair has failed. - **Procedure**: Similar to arthroscopic Bankart repair, but done through an open incision. The labrum is reattached to the glenoid, and the shoulder capsule is tightened. - **Recovery**: Requires a longer recovery period and potentially a more prolonged rehabilitation process compared to arthroscopic approaches.   **5. Capsular Shift or Plication**   - **Indication**: This is done for patients with generalized shoulder instability or when the shoulder capsule is stretched or redundant (not necessarily from a labral tear). - **Procedure**: The shoulder capsule is tightened (plicated) by folding and suturing the tissue to reduce excessive laxity, which helps stabilize the shoulder. - **Recovery**: A slower recovery process is required to allow the capsule to heal properly and restore shoulder function.   **6. Humeral Osteotomy**   - **Indication**: Rarely performed but can be considered in complex cases where altering the orientation of the humeral head is needed to address instability. - **Procedure**: The humeral head is surgically reshaped or repositioned to reduce the risk of dislocations. - **Recovery**: This is a major procedure with a longer recovery period and more intensive rehabilitation.   **7. Bristow Procedure**   - **Indication**: Similar to the Latarjet procedure, this is used in cases of significant bone loss or recurrent dislocation, though it is less commonly performed today. - **Procedure**: The coracoid process is transferred to the front of the glenoid, but unlike the Latarjet, it does not involve the entire muscle attachment, focusing mainly on the bone transfer. - **Recovery**: Rehabilitation is comparable to the Latarjet procedure.   **8. Bone Grafting**   - **Indication**: For patients with significant glenoid bone loss (usually more than 20% of the glenoid surface) due to recurrent dislocations or fractures. - **Procedure**: Bone grafts are taken from another part of the body (e.g., iliac crest) or from a cadaver and placed onto the glenoid to restore its normal anatomy and stability. - **Recovery**: Requires a longer healing time and focused rehabilitation to ensure the bone graft integrates with the surrounding structures.   **Summary of Surgical Considerations:**   - **Patient Factors**: The patient’s age, activity level, presence of bone loss, and frequency of dislocations guide the choice of surgery. - **Rehabilitation**: All surgeries require a dedicated rehabilitation program, which may last from 4 to 6 months, to restore full function, strength, and range of motion. - **Outcomes**: Generally, the surgical options listed above have good outcomes in terms of stabilizing the shoulder, but they differ in recovery time, complexity, and long-term prognosis.   These procedures aim to restore shoulder stability, prevent further dislocations, and allow patients to return to their daily activities or sports. | Surgical treatment options for **anterior shoulder instability** are often considered when non-surgical methods (such as physical therapy, rest, and bracing) fail to stabilize the shoulder or if the instability is recurrent, particularly in athletes or those with high physical demands. Here are the key surgical approaches:  **1. Arthroscopic Bankart Repair:**   - **Indication**: This is the most common surgical option for anterior shoulder instability, especially after recurrent dislocations. It's often used in cases of a torn **labrum** (the cartilage that deepens the shoulder socket). - **Procedure**: Minimally invasive, performed using small incisions and an arthroscope (a small camera). Surgeons reattach and repair the torn labrum to the glenoid (socket) using suture anchors. The torn **inferior glenohumeral ligament** may also be repaired. - **Pros**: Minimally invasive, less soft tissue damage, quicker recovery compared to open surgery. - **Cons**: Higher chance of recurrence compared to open procedures in certain populations (e.g., contact sports athletes).   **2. Open Bankart Repair:**   - **Indication**: This approach is sometimes preferred in patients with recurrent or complex instability, or in those who may not respond well to arthroscopic repair. - **Procedure**: A larger incision is made, and the surgeon directly accesses the joint to repair the labrum and ligaments. The same principles of restoring the torn labrum to its normal position are applied. - **Pros**: Lower risk of recurrent instability, especially in high-demand athletes. - **Cons**: Longer recovery time, more soft tissue damage.   **3. Latarjet Procedure (Coracoid Transfer):**   - **Indication**: Typically used when there is **significant bone loss** on the glenoid (socket) or after failed Bankart repairs. It is common in athletes or individuals with high physical demands. - **Procedure**: The surgeon transfers a portion of the **coracoid process** (a bone on the front of the shoulder blade) to the anterior glenoid to increase the bone surface and prevent further dislocations. The attached muscles also help stabilize the joint. - **Pros**: Highly effective for preventing recurrence, especially in patients with bone loss or multiple dislocations. - **Cons**: More invasive, with potential complications related to bone healing or hardware issues.   **4. Remplissage Procedure:**   - **Indication**: Often performed alongside a Bankart repair when there is a **Hill-Sachs lesion** (a dent in the humeral head caused by repeated dislocations). This lesion can contribute to recurrent dislocation. - **Procedure**: The surgeon fills in the defect on the humeral head by suturing the posterior capsule and infraspinatus tendon into the defect. This reduces the likelihood of the humeral head engaging with the glenoid rim. - **Pros**: Effective for addressing instability caused by Hill-Sachs lesions. - **Cons**: May limit shoulder motion slightly due to the change in shoulder mechanics.   **5. Capsular Shift or Capsulorrhaphy:**   - **Indication**: Used for patients with **multidirectional instability** or when the joint capsule is overly loose. - **Procedure**: The surgeon tightens and reinforces the capsule of the shoulder, reducing excessive laxity and preventing future dislocations. - **Pros**: Addresses capsular laxity, reducing the risk of future instability. - **Cons**: Longer recovery due to the need for capsule healing and potential motion limitations post-surgery.   **6. Bone Grafting Procedures:**   - **Indication**: Performed when there is **severe bone loss** in the glenoid or humeral head due to repeated dislocations. - **Procedure**: Bone grafts are taken from another part of the body (e.g., iliac crest) or using donor grafts to restore the shape of the glenoid or humeral head. - **Pros**: Helps restore the integrity of the shoulder joint in cases of significant bone loss. - **Cons**: More complex and involves additional healing time.   **Factors Influencing the Choice of Surgery:**   - **Level of physical activity**: Athletes may benefit more from procedures like the Latarjet. - **Bone loss**: Significant bone loss may require bony procedures (Latarjet or bone grafting). - **Age and tissue quality**: Older individuals with poor tissue quality may have a different surgical approach than younger, active patients. - **History of previous surgery**: Revisions of failed prior surgeries may require different techniques.   **Post-Surgical Rehabilitation:**   - Post-surgical physical therapy is crucial for regaining range of motion, strength, and stability. - The rehabilitation timeline depends on the procedure but typically involves 4-6 months for full recovery.   Consultation with an orthopedic surgeon is essential for selecting the most appropriate surgical treatment based on individual factors like activity level, extent of injury, and prior treatments. | This is for informational purposes only. For medical advice or diagnosis, consult a professional.  Surgical treatment for anterior shoulder instability typically involves arthroscopic procedures to repair or tighten the structures that stabilize the shoulder joint. The specific procedure depends on the nature and severity of the injury, but some common options include:   ([ref](https://pmc.ncbi.nlm.nih.gov/articles/PMC5646175/#:~:text=Anterior%20shoulder%20instability%20can%20be,as%20an%20individual%20case%20and))   - **Bankart repair:** This procedure repairs a tear in the labrum, a ring of cartilage that helps to stabilize the shoulder joint. ([ref](https://orthop.washington.edu/patient-care/articles/shoulder/bankart-repair-for-unstable-dislocating-shoulders.html#:~:text=Surgery%20to%20anatomically%20and%20securely,and%20improve%20function%20for%20active)) - **Capsular shift:** This procedure tightens the shoulder capsule, a layer of tissue that surrounds the shoulder joint.  ([ref](https://www.ortho.wustl.edu/content/Patient-Care/3141/Services/Shoulder-Elbow/Overview/Shoulder-Instability-Information/Surgical-Treatment-for-Shoulder-Instability.aspx#:~:text=Capsular%20shift%2D%20this%20procedure%20is,commonly%20performed%20using%20an%20arthroscope.)) - **Latarjet procedure:** This procedure involves transferring a piece of bone from the coracoid process (a bony prominence on the shoulder blade) to the glenoid (the socket of the shoulder joint) to provide additional stability. ([ref](https://www.medstarhealth.org/services/coracoid-transfer-latarjet-for-shoulder-instability#:~:text=In%20this%20open%20surgery%20technique,your%20shoulder%20blade%20to%20the))   The choice of surgical procedure will be made by the surgeon based on the individual patient's condition and needs. |
| **What are the risks and potential complications of anterior shoulder instability surgery?** | Anterior shoulder instability surgery, typically performed to repair a torn labrum, ligaments, or other soft tissues that contribute to shoulder instability, carries several risks and potential complications. These can be divided into general surgical risks and those more specific to shoulder surgery:  **General Surgical Risks**   1. **Infection**: As with any surgery, there is a risk of infection at the incision site or within the joint, although this is relatively uncommon. 2. **Bleeding**: Excessive bleeding during or after the procedure can occur, though it is generally minimal in arthroscopic surgeries. 3. **Anesthesia-related complications**: There is always a risk of complications from anesthesia, such as allergic reactions, breathing difficulties, or other adverse effects.   **Specific Risks and Complications of Anterior Shoulder Instability Surgery**   1. **Stiffness and Loss of Motion**: Shoulder stiffness or a reduction in the range of motion can occur after surgery. Scar tissue can form, limiting movement and flexibility. 2. **Recurrent Instability**: While surgery aims to restore shoulder stability, there is still a chance of the shoulder becoming unstable again. Recurrence of dislocations is more likely in people with certain risk factors (e.g., young athletes or high-contact sports participants). 3. **Nerve Injury**: Nerves around the shoulder joint, such as the axillary or suprascapular nerve, can be injured during surgery, leading to temporary or permanent numbness, weakness, or paralysis in the arm or shoulder. 4. **Arthritis**: Over time, the surgery may lead to the development of post-surgical arthritis in the shoulder joint due to cartilage damage or altered biomechanics. 5. **Hardware Complications**: In cases where screws, anchors, or other fixation devices are used, they may cause irritation, move, or loosen, requiring additional surgery to correct. 6. **Chronic Pain**: Some patients may experience persistent pain even after the surgery, which may stem from scar tissue, nerve damage, or improper healing. 7. **Failure of Repair**: In rare cases, the soft tissues (e.g., labrum, ligaments) may not heal adequately after surgery, leading to continued instability or a need for revision surgery. 8. **Capsular Contracture**: If the capsule (the connective tissue surrounding the joint) tightens excessively during healing, it can lead to restricted movement and stiffness. 9. **Blood Clots**: Though rare in shoulder surgeries, there is a small risk of developing blood clots (deep vein thrombosis) in the arms or other areas post-surgery.   **Patient-Specific Factors That Influence Risk**   - **Age**: Younger patients, especially athletes, may have a higher risk of re-injury or recurrence of instability. - **Activity Level**: Patients engaged in contact or overhead sports (e.g., rugby, basketball) may experience a higher likelihood of recurrence or complications. - **Type of Surgery**: Open surgery tends to have a higher risk of stiffness compared to minimally invasive arthroscopic surgery, but may provide greater stability in some cases.   **Managing and Minimizing Risks**  To minimize these risks, a structured rehabilitation program, adherence to post-operative care instructions, and proper communication with the surgeon and physical therapist are critical. | Surgery for **anterior shoulder instability**, often performed to stabilize the shoulder joint after dislocations or to repair damaged soft tissues, can be highly effective. However, as with any surgery, there are risks and potential complications. Here's a breakdown of what you might encounter:  **1. Infection**   - **Risk**: Infection can occur in the wound or deeper in the joint, though this is relatively rare (less than 1% in most cases). - **Prevention/Management**: Surgeons take precautions with sterile techniques, and patients are often given antibiotics to reduce the risk.   **2. Nerve Injury**   - **Risk**: Nerves around the shoulder, especially the **axillary nerve**, could be injured during surgery. Nerve damage can lead to **numbness**, **tingling**, or **weakness** in the shoulder or arm. - **Recovery**: Most nerve injuries are temporary, but in rare cases, they can be long-term or permanent.   **3. Stiffness and Loss of Motion**   - **Risk**: Post-surgery, the shoulder may become **stiff** or have a reduced **range of motion**, particularly if proper rehabilitation isn’t followed. - **Prevention**: Physical therapy is crucial for recovery to prevent this complication.   **4. Recurrent Instability or Dislocation**   - **Risk**: There is a small risk (5-10% in most cases) that the shoulder may become unstable again, especially in patients who participate in high-risk sports or activities. Factors like pre-existing ligament laxity can also contribute to this. - **Management**: Further surgery may be required if instability recurs.   **5. Failure of the Repair**   - **Risk**: The surgical repair could fail due to inadequate healing, improper fixation, or excessive stress on the shoulder post-surgery. - **Risk Factors**: This is more likely in people who return to high-contact sports too soon or have underlying tissue issues like poor quality of the ligaments or bone. - **Management**: If failure occurs, revision surgery might be necessary.   **6. Hardware Complications**   - **Risk**: For surgeries that use screws or anchors to secure tissues, the hardware can sometimes cause irritation, loosen, or migrate. - **Management**: In rare cases, additional surgery may be required to remove or adjust the hardware.   **7. Shoulder Weakness**   - **Risk**: Postoperative weakness, particularly with certain motions, can occur due to muscle atrophy, nerve injury, or incomplete recovery. - **Prevention**: A guided rehabilitation plan is essential to regain strength and function.   **8. Blood Clots (Deep Vein Thrombosis)**   - **Risk**: Although rare in shoulder surgeries, there is a small chance of developing a blood clot, particularly in the veins of the arm. - **Prevention**: Early mobilization and, in some cases, blood thinners may be used to reduce this risk.   **9. Scarring**   - **Risk**: Excessive **scarring** or **keloid formation** can occur at the surgical site, potentially causing discomfort or stiffness. - **Management**: Scars can usually be minimized through proper wound care and sometimes require additional treatments.   **10. Anesthetic Complications**   - **Risk**: As with any surgery involving general or regional anesthesia, complications related to anesthesia can occur. These might include allergic reactions, breathing issues, or prolonged recovery from anesthesia. - **Prevention/Management**: A thorough preoperative assessment by the anesthesiologist helps reduce this risk.   **Factors Increasing Risk of Complications:**   - **Age**: Older patients may have weaker tissues and longer recovery times. - **Repetitive Shoulder Trauma**: Athletes involved in contact sports or repetitive overhead movements are at higher risk of recurrent instability. - **Poor Tissue Quality**: Weak ligaments, poor bone quality, or prior surgeries can complicate recovery and increase the likelihood of complications.   **Success and Outcome**  Despite these risks, the success rate for anterior shoulder instability surgery is generally **high**, especially with proper post-op care. Rehabilitation plays a key role in minimizing complications and ensuring a successful return to daily activities or sports.  Would you like more information on specific recovery timelines or tailored rehab exercises following this surgery? | This is for informational purposes only. For medical advice or diagnosis, consult a professional.  Anterior shoulder instability surgery, while generally safe and effective, does carry some risks and potential complications. It's important to be aware of these before making a decision about surgery. ([ref](https://www.uhs.nhs.uk/departments/trauma-and-orthopaedics/shoulders/patient-information/anterior-stabilisation-of-the-shoulder))  **General Surgical Risks:**   - **Infection:** Although rare (generally under 1%), infections can occur at the incision site or within the shoulder joint itself. These may require antibiotics or further surgery to treat. ([ref](https://www.southtees.nhs.uk/resources/shoulder-stabilisation-surgery/)) - **Bleeding:** Some bleeding is normal during surgery, but excessive bleeding can lead to complications. - **Blood clots:** Blood clots can form in the legs or lungs after surgery, which can be dangerous. - **Nerve damage:** Nerves around the shoulder can be injured during surgery, leading to numbness, tingling, or weakness in the arm or hand. ([ref](https://www.ncbi.nlm.nih.gov/books/NBK538234/#:~:text=The%20axillary%20nerve%20can%20be,clock%20positions%2C%20respectively%2C%20in%20the)) - **Anesthesia complications:** Reactions to anesthesia can occur, although they are rare. ([ref](https://www.southtees.nhs.uk/resources/shoulder-stabilisation-surgery/))   **Specific Risks and Complications of Anterior Shoulder Instability Surgery:**   - **Recurrent instability:** The shoulder may become unstable again after surgery, requiring further treatment. This is more likely to occur in younger patients, athletes, and those with significant bone loss. ([ref](https://www.southtees.nhs.uk/resources/shoulder-stabilisation-surgery/#:~:text=Recurrent%20instability%3A,this%20may%20require%20further%20surgery.)) - **Stiffness:** Some stiffness in the shoulder is common after surgery, but it can sometimes become chronic and limit range of motion. - **Pain:** Pain is a normal part of the recovery process, but it can sometimes persist long-term. - **Hardware problems:** If implants such as screws or anchors are used, they can sometimes break or become loose, requiring further surgery to remove or replace them. - **Arthritis:** In some cases, surgery can accelerate the development of arthritis in the shoulder joint.  ([ref](https://pmc.ncbi.nlm.nih.gov/articles/PMC5359759/))   **Additional Considerations:**   - **Your overall health:** Certain medical conditions can increase the risk of complications from surgery. - **Your surgeon's experience:** Choosing an experienced surgeon can help to minimize the risks of surgery. - **Your rehabilitation:** Following your surgeon's instructions for rehabilitation is crucial for a successful outcome.   It's important to discuss the risks and potential complications of anterior shoulder instability surgery with your surgeon in detail. They can help you to understand the risks and benefits of surgery and make an informed decision about your treatment options.  **Disclaimer:** This information is not intended to be a substitute for professional medical advice. Always seek the advice of your physician or other qualified health provider with any questions you may have regarding a medical condition.   ([ref](https://www.vilantae.co.uk/live-support/)) ([ref](https://mdinteractive.com/terms-and-conditions)). |
| **What is the likelihood of recurrence after anterior shoulder instability treatment?** | The likelihood of recurrence after anterior shoulder instability treatment depends on various factors, including the type of treatment (surgical or non-surgical), patient characteristics (age, activity level), and the specific technique used in surgery. Here's a breakdown of the recurrence risk:  **Non-Surgical Treatment**  For patients who opt for non-surgical management of anterior shoulder instability (e.g., physical therapy, bracing):   - **High recurrence rates** are typically seen, particularly in younger and more active individuals. Studies suggest recurrence rates between **50% and 90%** in athletes, especially those involved in contact sports (e.g., rugby, football) or overhead activities (e.g., volleyball, swimming). - For less active or older patients, recurrence rates may be lower, but still significant, around **20% to 50%**.   **Surgical Treatment**  The likelihood of recurrence after surgery varies based on the type of procedure and patient factors:  **Arthroscopic Surgery (e.g., Arthroscopic Bankart Repair)**   - **Recurrence rates** range from **5% to 30%**, depending on the specific case and surgeon expertise. - Factors influencing higher recurrence rates include:   - **Age under 20–25 years**: Younger patients, especially under 20, have a higher recurrence rate, potentially reaching **15% to 30%**.   - **High activity levels**: Athletes in high-risk sports (contact or overhead) have a higher chance of recurrence (around **15% to 25%**).   - **Bone loss**: Significant bone loss in the shoulder (glenoid or humeral head) increases the risk of recurrent instability. In cases of glenoid bone loss over 20%, recurrence rates can be higher even after surgery.   **Open Surgery (e.g., Open Bankart Repair)**   - Open surgery tends to have slightly lower recurrence rates, ranging from **5% to 15%**. This approach may be used in cases with significant structural damage or when arthroscopic repair has failed. - Open procedures provide more stability but are associated with a slightly higher risk of post-operative stiffness.   **Latarjet Procedure**   - The **Latarjet procedure** is often performed in patients with significant bone loss or previous failed surgeries. It involves transferring a piece of bone (the coracoid) to the front of the shoulder to increase stability. - **Recurrence rates** after a Latarjet procedure are generally lower, around **2% to 5%**, making it a preferred option for patients with severe instability or bone loss.   **Factors Influencing Recurrence Risk**   1. **Age**: Younger patients (under 20–25 years old) are at higher risk, with recurrence rates as high as 30%, especially if returning to contact sports. 2. **Activity Level**: Athletes in high-demand sports are more likely to experience recurrent instability after surgery. Contact sports such as football, rugby, or wrestling increase the likelihood. 3. **Bone Loss**: The presence of bone loss in the glenoid or humeral head increases the chance of recurrence, particularly if not adequately addressed during surgery. 4. **Previous Surgeries**: Patients who have had failed previous surgeries are at greater risk of recurrent instability.   **Long-term Outlook**  While surgical intervention significantly reduces the risk of recurrence compared to non-surgical treatment, the likelihood of instability returning varies based on individual circumstances. Proper rehabilitation and adherence to post-operative care are essential for minimizing the risk of recurrence. | The likelihood of recurrence after treatment for anterior shoulder instability depends on several factors, including the type of treatment, the severity of the initial injury, the patient's activity level, and their age.  Here are the key factors influencing recurrence rates:   1. **Non-surgical (Conservative) Treatment:**    - **Recurrence rate**: For patients who undergo conservative management (such as physical therapy and strengthening exercises) without surgery, the recurrence rate can be quite high, especially in younger, active individuals. Studies report recurrence rates ranging from **30% to 90%**, with younger patients (especially those under 25) and athletes involved in contact sports being more likely to experience recurrent dislocations.    - **Why recurrence is high**: Conservative treatment may not adequately address the underlying damage to the joint, especially if there are significant structural injuries, like a Bankart lesion (tear of the labrum) or damage to the bone. 2. **Surgical Treatment (Arthroscopic or Open Repair):**    - **Arthroscopic Bankart Repair**: This is a common procedure for addressing labral tears in anterior shoulder instability. Recurrence rates after this surgery range from **5% to 20%** in the general population. Higher recurrence rates are often seen in younger patients and athletes involved in high-risk sports.    - **Open Bankart Repair**: Open surgery tends to have a slightly lower recurrence rate compared to arthroscopic surgery. Studies suggest recurrence rates between **3% to 10%**.    - **Latarjet Procedure**: This procedure is often used when there is significant bone loss. It has lower recurrence rates, generally less than **5%**, even in high-demand athletes. However, it is more invasive than Bankart repair and is typically used for recurrent instability cases or more severe injuries. 3. **Risk Factors for Recurrence:**    - **Age**: Younger patients (under 25) have a higher risk of recurrence after both non-surgical and surgical treatment.    - **Activity Level**: Athletes involved in contact sports (e.g., football, rugby) or overhead sports (e.g., tennis, baseball) are at increased risk for recurrent dislocations.    - **Bone loss**: Significant bone loss in the glenoid (socket) or humeral head increases the risk of recurrence, even after surgery. The Latarjet procedure is often recommended for cases of significant bone loss.    - **Initial injury severity**: The extent of the damage during the first dislocation (e.g., associated labral tears or fractures) can affect the likelihood of recurrence.   In summary:   - **Non-surgical treatment** has a high recurrence risk, especially in younger, active individuals. - **Surgical repair** generally offers better stability, with recurrence rates ranging from **5% to 20%**, depending on the procedure and patient-specific factors. The Latarjet procedure tends to offer the lowest recurrence risk - but is reserved for more complex cases.   Personal factors such as your age, activity level, and specific shoulder anatomy would greatly influence the likelihood of recurrence. Regular follow-up with your orthopedic specialist is key to monitoring the shoulder post-treatment. | This is for informational purposes only. For medical advice or diagnosis, consult a professional.  The likelihood of recurrence after anterior shoulder instability treatment can vary depending on several factors, including:   - **Age:** Younger patients, especially those under 20, have a higher risk of recurrence. ([ref](https://pubmed.ncbi.nlm.nih.gov/17079387/#:~:text=The%20younger%20male%20patients%20were,instability%20had%20this%20complication%20develop)) - **Activity level:** Athletes involved in contact sports have a higher risk of re-injury. - **Severity of the initial injury:** More severe injuries, such as those with bone loss, have a higher risk of recurrence. - **Type of treatment:** Surgical stabilization generally has a lower recurrence rate than non-surgical treatment. ([ref](https://pmc.ncbi.nlm.nih.gov/articles/PMC10375816/#:~:text=Open%20procedures%2C%20such%20as%20the,other%20approaches%2C%20such%20as%20an))   Overall, the recurrence rate after anterior shoulder instability treatment can range from 10% to 30%. However, with proper treatment and rehabilitation, the majority of patients can achieve good long-term outcomes and return to their desired activities.  It is important to discuss the risks and benefits of different treatment options with your doctor to make the best decision for your individual situation. |
| **What is a Bankart repair?** | A **Bankart repair** is a surgical procedure used to correct **shoulder instability**, particularly when there is a **Bankart lesion**. A Bankart lesion occurs when the labrum, a ring of cartilage that helps stabilize the shoulder joint, is torn due to **dislocation** or trauma. This tear often leads to recurrent shoulder dislocations, particularly in younger, active individuals.  **Anatomy and Injury:**   - The shoulder joint (glenohumeral joint) is a ball-and-socket joint, where the head of the humerus (arm bone) fits into the glenoid cavity of the scapula (shoulder blade). - The labrum deepens the socket, increasing stability. - When the shoulder dislocates, particularly **anteriorly** (front of the joint), the labrum can tear, causing a **Bankart lesion**.   **Bankart Repair Procedure:**   1. **Arthroscopic or Open Surgery**: The repair can be done arthroscopically (using small incisions and a camera) or through open surgery. 2. **Reattaching the Labrum**: The surgeon uses sutures and small anchors to reattach the torn labrum to the glenoid (socket), restoring its normal position and function. 3. **Restoring Joint Stability**: The procedure tightens the shoulder capsule and ligaments, which prevents future dislocations and improves overall stability of the shoulder joint.   **Indications:**   - Recurrent shoulder dislocations, particularly following an initial traumatic dislocation. - Shoulder instability that affects daily activities or sports.   **Recovery:**   - Patients typically require **immobilization** in a sling for a few weeks. - Physical therapy is a crucial part of rehabilitation, gradually improving range of motion and strength. - Full recovery can take several months, depending on the extent of the injury and individual healing.   Bankart repair is generally very successful, with high rates of return to sports and activities, though avoiding high-risk movements early in recovery is important to prevent re-injury. | A **Bankart repair** is a surgical procedure used to fix **shoulder instability**, specifically when there is a tear in the **labrum**, which is the soft cartilage rim that surrounds the shoulder socket (glenoid). This type of injury is often associated with **recurrent shoulder dislocations**, especially in younger, active individuals.  **Causes of Bankart Lesion:**  A **Bankart lesion** occurs when the shoulder joint dislocates and the labrum is torn. The labrum plays a crucial role in stabilizing the shoulder joint, so when it is damaged, the shoulder becomes prone to dislocations and instability.  **Bankart Repair Procedure:**   - **Open Bankart Repair**: A traditional method where an incision is made to access the shoulder joint, allowing direct visualization and repair of the torn labrum. - **Arthroscopic Bankart Repair**: A minimally invasive technique where small incisions are made, and a camera (arthroscope) is used to guide surgical instruments to repair the torn labrum with sutures or anchors.   **Goals of the Surgery:**  The goal is to reattach and stabilize the torn labrum to the glenoid (shoulder socket) so that the shoulder remains stable and does not dislocate again. This procedure restores the normal anatomy of the shoulder and prevents future episodes of dislocation.  **Indications for Bankart Repair:**   - Recurrent shoulder dislocations - Shoulder instability, especially when associated with labral tears - Failure of non-surgical treatments (like physical therapy)   **Recovery:**  Recovery typically involves:   - Immobilization of the shoulder for a few weeks using a sling - Gradual physical therapy to restore range of motion and strength - Full recovery may take 3-6 months, depending on the extent of the repair and patient activity levels.   The success rate of Bankart repairs is generally high, especially with modern arthroscopic techniques. However, rehabilitation is crucial for preventing future dislocations and achieving optimal outcomes. | This is for informational purposes only. For medical advice or diagnosis, consult a professional.  A Bankart repair is a surgical procedure to repair a Bankart lesion, which is a tear of the labrum (a ring of cartilage that helps stabilize the shoulder joint) that often occurs when the shoulder dislocates. ([ref](https://www.southtees.nhs.uk/resources/shoulder-stabilisation-surgery/))  The surgery involves reattaching the torn labrum to the bone using sutures or anchors. ([ref](https://my.clevelandclinic.org/health/diseases/bankart-lesion-glenoid-labrum-tear)) |
| **What is a Latarjet procedure?** | The **Latarjet procedure** is a type of shoulder surgery used to treat **recurrent shoulder dislocations** caused by bone loss or instability in the shoulder joint, specifically at the front of the glenoid (the socket part of the shoulder joint). It's named after French surgeon **Michel Latarjet**, who first described the technique in the 1950s.  **The Procedure:**  In the Latarjet procedure, the surgeon removes a portion of the **coracoid process**, which is a small hook-like bone in the shoulder, along with its attached tendon, and transfers it to the **front of the glenoid**. This bone transfer helps:   1. **Increase stability**: The bone graft adds to the bony surface area of the glenoid, making the socket deeper and preventing the head of the humerus (the upper arm bone) from dislocating. 2. **Reinforce soft tissues**: The attached tendon (the coracoacromial ligament) helps reinforce the front of the shoulder and prevents further dislocations.   **Indications for Latarjet Procedure:**  The Latarjet procedure is typically recommended when:   - There has been a significant amount of **bone loss** in the glenoid, often due to multiple dislocations. - Other stabilization techniques, such as soft tissue repairs (e.g., the **Bankart repair**), are insufficient or have failed. - The patient has **chronic shoulder instability** despite non-surgical treatments.   **Recovery:**  Recovery from a Latarjet procedure involves a period of immobilization (usually with a sling) followed by physical therapy to regain strength and range of motion. Full recovery can take several months, and most patients are able to return to their previous activities, including sports, with a reduced risk of future dislocations.  It is a technically demanding surgery but has a high success rate for improving shoulder stability. | The **Latarjet procedure** is a surgical technique used primarily to treat recurrent shoulder dislocations or shoulder instability, especially when there is significant bone loss in the glenoid (the socket part of the shoulder joint) or when other soft tissue repairs have failed. It is named after French surgeon Michel Latarjet, who first described the procedure.  **Key Elements of the Latarjet Procedure:**   1. **Bone Transfer**: The procedure involves transferring a small portion of the coracoid process (a bone in the shoulder blade) along with its attached tendons to the front of the glenoid. This helps increase the size of the glenoid and prevent future dislocations. 2. **Tendon Reinforcement**: The tendons attached to the coracoid are used to reinforce the shoulder joint by acting as a dynamic sling that stabilizes the shoulder, particularly during arm movements. 3. **Stabilizing Mechanism**:    - **Bone Block**: By moving the coracoid to the front of the glenoid, the bony portion prevents the shoulder from dislocating forward.    - **Muscle Sling**: The tendons attached to the coracoid, like the subscapularis, help provide additional support and stability.   **Indications for the Latarjet Procedure:**   - **Recurrent Shoulder Dislocations**: Particularly when there is damage to the glenoid or when soft tissue procedures like the Bankart repair are insufficient. - **Bony Defects**: Significant bone loss or fracture of the glenoid rim due to repeated dislocations. - **Failed Previous Surgery**: If previous procedures aimed at stabilizing the shoulder have failed.   **Recovery and Rehabilitation:**   - **Immobilization**: Post-operatively, the shoulder is usually immobilized in a sling for a few weeks to allow healing. - **Physical Therapy**: Gradual physical therapy is started to regain range of motion, strength, and stability. Full recovery and return to sports can take around 4-6 months, depending on the individual case.   **Potential Risks:**   - Infection, nerve injury, or failure of the bone to heal. - Persistent shoulder stiffness or weakness if rehabilitation is not done properly.   The Latarjet procedure is highly effective in reducing recurrent shoulder instability and is commonly used for athletes and active individuals who put significant stress on their shoulders. | This is for informational purposes only. For medical advice or diagnosis, consult a professional.  A Latarjet procedure is a surgical procedure used to treat recurrent shoulder dislocations, typically caused by bone loss or a fracture of the glenoid .([ref](https://pmc.ncbi.nlm.nih.gov/articles/PMC3235430/#:~:text=1.1.,recurrent%20dislocation%20of%20the%20shoulder.)) The procedure ([ref](https://www.ieyenews.com/a-regional-first-as-cayman-islands-surgeons-repair-rugby-players-shoulder/)) involves transferring a piece of bone with an attached tendon to the front of the glenoid to stabilize the joint.  ([ref](https://www.medstarhealth.org/services/coracoid-transfer-latarjet-for-shoulder-instability#:~:text=In%20this%20open%20surgery%20technique,your%20shoulder%20blade%20to%20the))  Here's a breakdown of the procedure:   1. **Bone graft:** A piece of bone, usually taken from the coracoid process (a small hook-like structure on the shoulder blade), is removed. ([ref](https://www.windsorupperlimb.com/procedures/shoulder-procedures/open-stabilisation-latarjet-procedure)) 2. **Transfer:** This bone graft is then transferred to the front of the glenoid (the socket part of the shoulder joint) and fixed in place with screws. ([ref](https://www.londonarmdoc.com/arthroscopic-latarjet-for-shoulder-instability-shoulder-surgeon-london/#:~:text=Hence%2C%20during%20the%20procedure%2C%20rather,the%20glenoid%20and%20fixed%20in)) 3. **Tendon sling:** The tendon attached to the bone graft acts as a sling, providing additional support and stability to the shoulder joint. ([ref](https://www.londonarmdoc.com/arthroscopic-latarjet-for-shoulder-instability-shoulder-surgeon-london/#:~:text=The%20bone%20graft%20and%20its,the%20function%20of%20the%20labrum.))   The Latarjet procedure is generally considered a safe and effective way to treat recurrent shoulder dislocations, particularly in cases with significant bone loss. However, it is important to note that like any surgical procedure, there are potential risks and complications associated with it.   ([ref](https://pmc.ncbi.nlm.nih.gov/articles/PMC4437927/#:~:text=A%2020%2Dyear%20follow%2Dup,only%205.9%25%2C%20which%20indicated%20that))  If you are considering a Latarjet procedure, it is important to discuss the risks and benefits with your surgeon. They will be able to help you determine if the procedure is right for you. |
| **What is a Remplissage procedure?** | The **Remplissage procedure** is a surgical technique used to treat **shoulder instability**, particularly in cases of recurrent shoulder dislocations that involve a **Hill-Sachs lesion**. A Hill-Sachs lesion is a bone defect in the humeral head (the ball of the shoulder joint) that occurs when it hits the edge of the glenoid (the socket) during a shoulder dislocation.  The term "Remplissage" comes from French, meaning "to fill in" or "to fill up." The procedure involves filling the Hill-Sachs defect with the posterior (backside) capsule of the shoulder and the **infraspinatus tendon**, one of the rotator cuff muscles. The goal is to prevent the humeral head from engaging with the edge of the glenoid, thereby reducing the risk of future dislocations.  **How the Remplissage Procedure Works:**   1. **Arthroscopic Surgery**: The procedure is typically done arthroscopically, meaning small incisions are made, and a camera and tools are inserted into the shoulder joint. 2. **Filling the Defect**: The posterior capsule and infraspinatus tendon are sutured or anchored into the Hill-Sachs lesion. This effectively "fills" the bone defect with soft tissue, reducing the chance of it catching or engaging with the glenoid rim. 3. **Concurrent Bankart Repair**: Often, a Remplissage procedure is done in combination with a **Bankart repair**, which addresses a labral tear on the front of the shoulder. A Bankart tear commonly occurs alongside the Hill-Sachs lesion in cases of anterior shoulder instability.   **Indications for the Remplissage Procedure:**   - Patients with recurrent anterior shoulder dislocations. - The presence of a moderate to large **Hill-Sachs lesion**. - **Engaging Hill-Sachs lesion**: This means that the bone defect in the humeral head catches or engages with the glenoid rim during arm movements, leading to recurrent dislocations.   **Benefits:**   - Reduces the risk of future shoulder dislocations by addressing both the soft tissue (labral tear) and the bony defect. - Minimally invasive and usually performed arthroscopically. - Can be done alongside other stabilizing procedures like a Bankart repair.   **Potential Downsides:**   - Postoperative loss of some external rotation due to tension on the infraspinatus muscle. - Like any surgical procedure, there are risks of complications, such as infection, stiffness, or incomplete relief from instability.   In summary, the Remplissage procedure is a valuable technique for managing complex cases of shoulder instability, especially when a Hill-Sachs lesion is contributing to recurrent dislocations. | The **Remplissage procedure** is a surgical technique used to treat **shoulder instability** caused by a condition known as a **Hill-Sachs lesion**. A Hill-Sachs lesion is a dent or defect in the **humeral head** (the ball part of the shoulder joint) that occurs when the shoulder repeatedly dislocates or is unstable.  In a shoulder dislocation, the ball of the upper arm bone can hit the edge of the socket, causing damage to the humeral head. This damage can then contribute to recurring shoulder dislocations.  **How the Remplissage Procedure Works:**  The term "remplissage" comes from the French word meaning "to fill." In this procedure:   1. The surgeon arthroscopically "fills" or "plugs" the Hill-Sachs defect by attaching part of the **infraspinatus tendon** (one of the rotator cuff muscles) and the **joint capsule** into the humeral head defect. 2. By doing this, the damaged area of the humeral head is filled with soft tissue, reducing the risk of the shoulder dislocating again. 3. This technique effectively converts the area from an intra-articular defect (inside the joint) to an extra-articular one (outside the joint), reducing the risk of the defect engaging with the glenoid (socket) and causing further dislocations.   **When is Remplissage Recommended?**  It is typically recommended for patients who:   - Have a significant Hill-Sachs lesion contributing to shoulder instability. - Suffer from recurrent shoulder dislocations or instability, particularly when the shoulder moves into certain positions. - Have failed conservative treatments or other types of shoulder stabilization surgeries.   The **Remplissage** procedure is often performed alongside a **Bankart repair**, where the damaged labrum (cartilage in the shoulder socket) is also repaired to improve overall shoulder stability.  **Postoperative Care and Recovery:**  After the procedure, physical therapy is usually necessary to regain shoulder strength and mobility. The typical recovery time can range from **4-6 months**, depending on the extent of the injury and how well the patient follows the rehabilitation plan. | This is for informational purposes only. For medical advice or diagnosis, consult a professional.  A remplissage procedure is a surgical technique used to treat shoulder instability, particularly when there is a Hill-Sachs lesion present.   ([ref](https://pmc.ncbi.nlm.nih.gov/articles/PMC8243109/#:~:text=The%20remplissage%20procedure%2C%20which%20means,of%20the%20posterior%20capsule%20and))  Here's a breakdown of the procedure:  **What is a Hill-Sachs lesion?**  A Hill-Sachs lesion is a compression fracture on the head of the humerus (upper arm bone) that occurs due to repeated shoulder dislocations. When the shoulder dislocates, the humeral head can impact against the glenoid (shoulder socket), resulting in this bony defect. ([ref](https://my.clevelandclinic.org/health/diseases/24304-hill-sachs-lesion#:~:text=Hill%2DSachs%20lesions%20are%20caused%20when%20your%20shoulder%20is%20dislocated.&text=Hill%2DSachs%20lesions%20are%20a,you%20experience%20an%20anterior%20glenohumeral))  **Why is remplissage necessary?**  Shoulder instability can significantly impact an individual's quality of life, leading to pain, limited mobility, and recurrent dislocations. When a Hill-Sachs lesion is present, the likelihood of recurrent dislocation is higher. ([ref](https://my.clevelandclinic.org/health/diseases/24304-hill-sachs-lesion#:~:text=If%20you%20have%20a%20large,another%20dislocation%20in%20the%20future.)) Remplissage addresses this by filling the defect with the infraspinatus tendon, effectively preventing the humeral head from engaging the glenoid rim during certain movements.  ([ref](https://www.grantgarciamd.com/remplissage.html))  **How is the procedure performed?**  Remplissage is typically performed arthroscopically, meaning it involves small incisions and specialized instruments. ([ref](https://www.physio-pedia.com/Remplissage_Procedure)) The surgeon detaches the infraspinatus tendon and advances it into the humeral head defect, effectively filling the lesion. ([ref](https://aoj.amegroups.org/article/view/3873/html#:~:text=The%20technique%20involves%20the%20use,humeral%20lesion%20to%20prevent%20continued)) The tendon is then secured in place using sutures or anchors.  ([ref](https://www.shoulderdoc.co.uk/article/1300#:~:text=Sutures%20are%20passed%20through%20the,parachute%20technique'%2C%20hence%20successfully%20filling))  **What are the benefits of remplissage?**  Remplissage has shown promising results in reducing shoulder instability and minimizing the risk of future dislocations. ([ref](https://www.grantgarciamd.com/remplissage.html)) Studies have demonstrated a significant decrease in recurrent instability and improved patient satisfaction rates following the procedure. ([ref](https://pmc.ncbi.nlm.nih.gov/articles/PMC9042916/))  **Important considerations:**   - **Individual outcomes may vary:** The success of the procedure can depend on the severity of shoulder instability and other factors. - **Rehabilitation is crucial:** Following the postoperative rehabilitation program is essential for optimizing results.  ([ref](https://ijspt.scholasticahq.com/article/123481-rehabilitation-protocol-variability-following-arthroscopic-bankart-repair-and-remplissage-for-management-of-anterior-shoulder-instability-a-systemati#:~:text=Therefore%2C%20post%2Doperative%20rehabilitation%20plays,as%20facilitating%20optimal%20healing%20of))   If you have any concerns about shoulder instability or the remplissage procedure, it's important to consult with an orthopedic surgeon who specializes in shoulder conditions. |
| **What are the indications and contraindications for Bankart repair?** | **Bankart repair** is a surgical procedure primarily performed to address recurrent anterior shoulder dislocation, particularly when a **Bankart lesion** (damage to the anterior-inferior labrum and associated capsule) is present. The procedure aims to restore stability to the shoulder by repairing and reattaching the torn labrum and tightening the surrounding ligaments.  **Indications for Bankart Repair**  Bankart repair is indicated in the following situations:   1. **Recurrent anterior shoulder dislocations**:    - Patients with a history of recurrent anterior dislocations following an initial traumatic shoulder injury.    - Usually occurs in younger, active individuals (commonly athletes) or patients with significant physical demands on their shoulders. 2. **Bankart lesion**:    - Damage to the anteroinferior glenoid labrum (typically due to a traumatic event).    - Often seen on imaging studies (MRI, arthroscopy) confirming labral detachment from the glenoid rim. 3. **Capsular laxity**:    - Patients with shoulder instability due to looseness of the joint capsule along with a Bankart lesion.    - These patients typically have excessive shoulder mobility. 4. **Failure of conservative management**:    - Patients who have tried non-surgical management (physical therapy, strengthening exercises, activity modification) but continue to experience recurrent instability or dislocation. 5. **Active lifestyle**:    - Patients involved in high-risk or overhead activities (sports like swimming, baseball, tennis, gymnastics) who are at risk of recurrent shoulder dislocations. 6. **Younger patients**:    - Patients typically younger than 40 years old are more likely to experience recurrent dislocations and may benefit from surgical stabilization. 7. **Presence of associated injuries**:    - Other shoulder pathology (such as Hill-Sachs lesions or glenoid bone loss) that may contribute to shoulder instability and require surgical correction.   **Contraindications for Bankart Repair**  Bankart repair is contraindicated or not recommended in the following situations:   1. **Multidirectional shoulder instability**:    - Patients with generalized shoulder instability not limited to anterior dislocations (often due to global laxity of the capsule), especially those with no clear labral tear.    - These patients might benefit more from a capsular shift or a different procedure addressing the whole capsule. 2. **Ehlers-Danlos syndrome or other connective tissue disorders**:    - These patients often have excessive joint laxity, and the success rate of a Bankart repair may be lower due to the inherent collagen and tissue abnormalities. 3. **Severe glenoid bone loss**:    - If more than 25% of the glenoid bone is damaged or missing (glenoid rim fracture or attritional bone loss), a Bankart repair alone may be insufficient.    - These cases may require a bony augmentation procedure such as a Latarjet procedure. 4. **Inadequate tissue quality**:    - In cases of poor tissue quality (e.g., from chronic tears or degenerative conditions), the Bankart repair may fail due to an inability to achieve secure fixation of the labrum or capsule. 5. **Advanced age**:    - Patients older than 40 years with shoulder dislocations may have different pathologies (e.g., rotator cuff tears) that are more significant contributors to instability. In such cases, a different surgical approach may be needed. 6. **Non-compliance with post-operative rehabilitation**:    - Patients who are unlikely to adhere to post-surgical rehabilitation protocols, which are crucial for successful recovery and maintenance of shoulder stability, should be considered poor candidates for surgery. 7. **Infection or inflammatory conditions**:    - Active infection in or around the shoulder joint or systemic inflammatory conditions that could impede healing are contraindications for surgical intervention.   By understanding these indications and contraindications, surgeons can decide when Bankart repair is the most appropriate option for shoulder instability management. | A **Bankart repair** is a surgical procedure primarily used to treat recurrent shoulder instability due to a **Bankart lesion**, which involves a tear of the anterior-inferior labrum of the shoulder joint. The key indications for Bankart repair include:   1. **Recurrent Shoulder Dislocations**: Patients experiencing repeated shoulder dislocations, especially in the anterior direction, often require surgical stabilization. 2. **Bankart Lesion**: Imaging (like MRI or arthroscopy) reveals a tear in the labrum, specifically at the anterior-inferior region of the glenoid. 3. **Young, Active Patients**: Younger patients, particularly athletes or individuals involved in activities that place the shoulder under stress (e.g., contact sports, overhead throwing), are more likely to benefit from surgery to prevent further instability. 4. **Failed Conservative Management**: If non-operative treatments (such as physical therapy, bracing, and activity modification) fail to restore stability and prevent dislocation, surgical intervention may be considered. 5. **Associated Pathologies**: Bankart repair may also be indicated in patients with Hill-Sachs lesions (indentation fracture of the humeral head) or minor bone loss, provided that these pathologies are not severe. 6. **High-Risk Occupational or Lifestyle Factors**: Individuals whose work (e.g., manual labor) or lifestyle (e.g., extreme sports) demands shoulder stability may opt for surgery even after a single dislocation to avoid recurrent instability.   **Contraindications for Bankart Repair:**  Several factors can make Bankart repair less suitable or contraindicated:   1. **Multidirectional Instability**: If a patient has generalized or multidirectional shoulder instability (instability in more than one direction), Bankart repair alone may not be sufficient. These cases may require alternative or additional procedures like capsular plication. 2. **Significant Bone Loss**: Substantial bone loss on the glenoid rim (>20-25%) or the humeral head may not be addressed adequately with Bankart repair alone. In such cases, alternative procedures like a **Latarjet** or bone grafting might be more appropriate. 3. **Severe Hill-Sachs Lesion**: If there is a large Hill-Sachs lesion (engaging Hill-Sachs lesion), Bankart repair alone may not prevent continued shoulder instability. Addressing both the humeral defect and the labral tear may be necessary. 4. **Elderly Patients**: Older individuals with decreased tissue quality or shoulder dislocations caused by rotator cuff tears might not benefit from Bankart repair. Soft tissue repair may not hold as well in this population due to age-related degenerative changes. 5. **Poor Tissue Quality**: Patients with connective tissue disorders (e.g., Ehlers-Danlos syndrome) or poor-quality labral tissue may not have sufficient tissue for a successful repair. 6. **Infection or Uncontrolled Systemic Illness**: Any active infection around the shoulder or serious systemic illness would be a contraindication until resolved. 7. **Lack of Patient Compliance**: Post-operative rehabilitation is crucial to successful outcomes after a Bankart repair. Patients who are unlikely to comply with rehabilitation protocols or restrictions might have a higher risk of failure.   These indications and contraindications guide the decision-making process to ensure that Bankart repair is performed on the appropriate candidates and delivers the best possible outcomes. | This is for informational purposes only. For medical advice or diagnosis, consult a professional.  Here's a breakdown of the indications and contraindications for Bankart repair, keeping in mind the year is 2025:  **Indications**  Bankart repair is primarily indicated for patients with recurrent anterior shoulder instability, meaning their shoulder has dislocated or subluxed multiple times. This instability is often caused by a Bankart lesion, which is a tear of the glenoid labrum (a ring of cartilage that helps stabilize the shoulder joint) and the inferior glenohumeral ligament.  **Specific indications include:**   - **Recurrent anterior shoulder instability:** This is the most common indication. Patients experience repeated episodes of their shoulder "giving way" or dislocating, often with minimal trauma. - **Traumatic anterior shoulder dislocation:** In some cases, Bankart repair may be considered after a first-time traumatic dislocation, especially in young athletes or individuals with high demands on their shoulder. ([ref](https://www.e-emj.org/archive/view_article?pid=emj-47-2-21)) - **Failed conservative treatment:** If physical therapy and other non-surgical treatments haven't resolved the instability, surgery may be necessary. ([ref](https://www.dukehealth.org/treatments/orthopaedics/shoulder-dislocation-and-instability#:~:text=If%20nonsurgical%20treatments%20and%20physical,dislocations%20and%20return%20you%20to))   **Contraindications**  Bankart repair is not suitable for all patients with shoulder instability. Contraindications include: ([ref](https://www.shoulderdoc.co.uk/section/870))   - **Multidirectional instability:** If the shoulder is unstable in multiple directions, Bankart repair alone may not be sufficient. - **Significant bone loss:** If there is substantial bone loss in the glenoid (socket) or humeral head (ball) due to previous dislocations or fractures, additional procedures like bone grafting may be required. - **Large Hill-Sachs lesion:** A Hill-Sachs lesion is a dent in the humeral head that can occur during a dislocation. If it's large and engaging (meaning it catches on the glenoid), Bankart repair alone may not be enough.   ([ref](https://pmc.ncbi.nlm.nih.gov/articles/PMC8418586/#:~:text=A%20Hill%2DSachs%20lesion%20is,an%20anterior%20SD%20%5B6%5D.)) - **Active infection:** Surgery should be postponed until any active infection is resolved. - **Medical conditions:** Certain medical conditions that increase the risk of complications from surgery may be considered relative contraindications.   **Additional Considerations**   - **Patient age and activity level:** Younger patients and those involved in contact sports or overhead activities may be more likely to benefit from surgery. ([ref](https://www.physio-pedia.com/Bankart_lesion)) - **Patient compliance:** Successful outcomes depend on the patient's adherence to post-operative rehabilitation protocols.  ([ref](https://pmc.ncbi.nlm.nih.gov/articles/PMC5958453/#:~:text=Background%3A,and%20participation%20in%20physical%20therapy.))   **It's important to consult with an orthopedic surgeon to determine if Bankart repair is the right treatment option for your specific situation.** |
| **What are the indications and contraindications for the Latarjet procedure?** | The **Latarjet procedure** is a surgical technique used primarily to treat recurrent shoulder instability, especially when there is significant bone loss in the glenoid (socket of the shoulder) or a large Hill-Sachs lesion (a compression fracture of the humeral head). It involves transferring the coracoid process (a small bone on the front of the shoulder blade) along with its attached muscles and tendons to the front of the glenoid to increase stability by enlarging the glenoid and providing a dynamic sling effect.  **Indications for the Latarjet procedure:**   1. **Recurrent anterior shoulder instability**:    - **Chronic shoulder dislocations** that do not respond well to conservative treatments or previous surgeries (e.g., arthroscopic stabilization).    - Patients with repeated episodes of dislocation, especially during high-risk activities. 2. **Glenoid bone loss**:    - Considered when there is a significant bone loss of more than 20–25% of the glenoid surface area. Bone loss decreases the effectiveness of soft-tissue stabilization procedures. 3. **Hill-Sachs lesion**:    - A large or "engaging" Hill-Sachs lesion, which is a defect in the humeral head that engages with the rim of the glenoid, increasing the risk of dislocation. 4. **Failed previous stabilization surgeries**:    - When patients have failed other stabilization surgeries (e.g., Bankart repair) due to glenoid or humeral bone loss. 5. **Contact or overhead sports participation**:    - Patients who participate in contact sports (e.g., rugby, football) or overhead sports (e.g., tennis, swimming) are often good candidates due to the demands these activities place on shoulder stability. 6. **High-risk groups**:    - Individuals with high physical demands or those in physically demanding occupations, such as military personnel, firefighters, or athletes.   **Contraindications for the Latarjet procedure:**   1. **Minimal or no glenoid bone loss**:    - In cases of shoulder instability without significant bone loss, less invasive procedures, such as arthroscopic Bankart repair, may be more appropriate. 2. **Infection or active shoulder sepsis**:    - Any infection in or around the shoulder joint would preclude the procedure until treated. 3. **Severe rotator cuff pathology**:    - The Latarjet procedure is not suitable for patients with significant rotator cuff tears or pathology, as the procedure focuses on anterior stabilization and requires intact soft tissue to work effectively. 4. **Severe glenohumeral arthritis**:    - In patients with advanced arthritis of the glenohumeral joint, a joint replacement may be more appropriate. 5. **Inadequate bone stock or poor bone quality**:    - Patients with conditions such as severe osteoporosis, where the transferred coracoid or screws might not achieve good fixation, may not be good candidates. 6. **Young patients (still growing)**:    - Skeletally immature patients, such as children or adolescents with open growth plates, may not be ideal candidates for the Latarjet procedure because of the potential effects on bone growth and development. 7. **Patient noncompliance or inability to adhere to postoperative rehabilitation**:    - If a patient is unlikely to comply with the necessary postoperative rehabilitation or has issues that would complicate recovery, they may not be a good candidate for the procedure.   The Latarjet procedure is a highly effective and specialized surgery with good outcomes when performed for the correct indications, but it is not without risks, especially when contraindications are present. | The **Latarjet procedure** is a surgical technique used primarily to address recurrent shoulder dislocations and instability, often related to significant bone loss. It involves transferring a section of the coracoid process to the glenoid (shoulder socket) to increase stability. Here are the **indications** and **contraindications** for the Latarjet procedure:  **Indications for the Latarjet procedure:**   1. **Recurrent anterior shoulder dislocation**:    - This is the primary indication, especially in cases where conservative treatments have failed, and the patient experiences frequent shoulder dislocations. 2. **Glenoid bone loss**:    - The procedure is particularly useful when there is significant (usually > 20-25%) bone loss in the anterior part of the glenoid due to repeated dislocations, making soft-tissue repairs like the Bankart procedure inadequate. 3. **Engaging Hill-Sachs lesion**:    - A large, engaging Hill-Sachs lesion (posterolateral humeral head compression fracture) that interacts with the glenoid rim during shoulder movements may warrant the Latarjet procedure if it leads to instability. 4. **Failed previous stabilization surgeries**:    - Patients who have undergone procedures like the Bankart repair but continue to experience dislocations or instability may be candidates for the Latarjet, as it provides additional bony support. 5. **Contact or collision sports**:    - Athletes participating in high-risk activities such as rugby, football, or martial arts, where shoulder impact is frequent, may require a more robust stabilization that the Latarjet offers. 6. **Hyperlaxity** with instability:    - Patients with generalized ligamentous laxity (loose joints) and recurrent instability that makes soft-tissue repairs insufficient may benefit from the additional bony support provided by the Latarjet.   **Contraindications for the Latarjet procedure:**   1. **Absence of significant bone loss**:    - In cases of shoulder instability without significant glenoid or humeral bone loss, less invasive procedures like the arthroscopic Bankart repair are often preferred. 2. **Rotator cuff tears**:    - Significant, irreparable rotator cuff pathology may make the Latarjet procedure less favorable, as the surgery does not directly address rotator cuff issues. 3. **Severe arthritis**:    - If the patient has advanced glenohumeral arthritis, the Latarjet procedure may not be appropriate since the bony transfer does not address joint degeneration. 4. **Infection or active inflammation**:    - Any infection or active inflammation in or around the shoulder joint would be a contraindication, as this increases the risk of postoperative complications. 5. **Patient noncompliance**:    - The success of the Latarjet procedure depends on postoperative rehabilitation and patient compliance. If a patient is unlikely to follow the necessary recovery protocols, the outcomes may be compromised, making it less suitable. 6. **Severe glenoid dysplasia**:    - In cases of significant congenital or developmental abnormalities of the glenoid (such as dysplasia), where anatomical reconstruction might not be feasible, alternative surgical interventions may be considered. 7. **Poor general health or anesthesia risk**:    - Patients with severe systemic health conditions that pose high risks for surgery or anesthesia complications should be carefully evaluated before considering the Latarjet procedure.   **Summary:**  The **Latarjet procedure** is an effective option for patients with recurrent shoulder dislocations, significant bone loss, or failed previous stabilization surgeries. It is contraindicated in cases where bone loss is minimal, when there are coexisting pathologies (like rotator cuff tears or arthritis), or if the patient is medically unfit for surgery. Proper patient selection is critical for the success of the procedure. | This is for informational purposes only. For medical advice or diagnosis, consult a professional.  Here are the indications and contraindications for the Latarjet procedure, a surgical procedure used to treat recurrent anterior shoulder instability:  **Indications**   - Recurrent anterior shoulder dislocations or subluxations (partial dislocations) ([ref](https://www.scielo.br/j/aob/a/JsMkx4qTPfgKmrGfpdtnksk/#:~:text=Conclus%C3%A3o%3A%20A%20cirurgia%20de%20Latarjet,do%20ombro%2C%20possibilitando%20melhora%20funcional)) - Significant glenoid bone loss (the socket of the shoulder joint) ([ref](https://pmc.ncbi.nlm.nih.gov/articles/PMC7453470/#:~:text=It%20had%20been%20shown%20before,indication%20for%20the%20Latarjet%20procedure.)) - Failed previous shoulder stabilization surgery  ([ref](https://openorthopaedicsjournal.com/VOLUME/13/PAGE/272/FULLTEXT/#:~:text=The%20Latarjet%20procedure%20is%20used,or%20failed%20previous%20stabilisation%20surgery.)) - Participation in high-risk sports or activities that place significant stress on the shoulder joint   **Contraindications**   - Active infection in the shoulder joint - Insufficient bone stock for graft fixation - Irreparable rotator cuff tear - Neurological conditions that may affect shoulder stability (e.g., epilepsy) - Voluntary shoulder instability (ability to dislocate the shoulder at will)   It is important to note that the decision to proceed with the Latarjet procedure is made on a case-by-case basis after a thorough evaluation by an orthopedic surgeon. |
| **What is the expected recovery time after surgery for anterior shoulder instability?** | The expected recovery time after surgery for anterior shoulder instability (often done through arthroscopy or open surgery) depends on several factors, including the type of procedure, the severity of the injury, and the patient's overall health. In general:   1. **Initial recovery (1-2 weeks):** After surgery, the arm is usually immobilized in a sling for 2 to 6 weeks to protect the shoulder and allow the tissue to heal. Pain and discomfort are typically managed with medication during this period. 2. **Rehabilitation phase (6-12 weeks):**    - **Weeks 3-6:** Patients start physical therapy, focusing on gentle range-of-motion exercises, without stressing the repaired structures.    - **Weeks 6-12:** Strengthening exercises are introduced to regain shoulder stability and muscle strength. 3. **Full recovery (3-6 months):**    - **By 3 months:** Most patients can return to light daily activities and some non-contact sports.    - **By 6 months:** Patients can typically resume more strenuous activities, including contact sports or heavy lifting, assuming strength and mobility have returned. Full recovery of strength and motion can take up to 6 months or longer, depending on the individual. 4. **Return to sports (6-12 months):** Athletes or those involved in high-demand activities may take 6-12 months to fully return to their previous level of activity, especially in contact sports.   Rehabilitation and adhering to post-operative instructions are critical for a successful recovery and preventing re-injury. Always follow the guidance of your surgeon and physical therapist for a more tailored recovery plan. | The recovery time after surgery for anterior shoulder instability can vary depending on several factors, including the type of surgical procedure performed (open or arthroscopic), the severity of the instability, the patient's overall health, and adherence to post-operative rehabilitation. However, here are some general guidelines:  **1. Immobilization Period (0-6 weeks):**   - **Sling use:** Typically, the arm is placed in a sling for the first 3-6 weeks after surgery to protect the repair and allow the tissues to heal. - **Restricted movement:** Range of motion (ROM) exercises are generally limited during this period to avoid stressing the repair site.   **2. Early Rehabilitation (6-12 weeks):**   - **Physical therapy:** Passive and active-assisted range of motion exercises begin to restore movement to the shoulder gradually. - **Strengthening:** Light strengthening exercises start, but heavier or more intense activities are still avoided.   **3. Intermediate Phase (12-16 weeks):**   - **Progressive strengthening:** Resistance exercises are incorporated to strengthen the rotator cuff and shoulder stabilizers. - **Full ROM:** By this stage, most patients should regain nearly full range of motion, though some may still have limitations.   **4. Advanced Rehabilitation (4-6 months):**   - **Return to daily activities:** Patients can begin more functional activities and work toward returning to sports or physically demanding tasks. - **Sport-specific training:** For athletes, sport-specific drills and conditioning are gradually reintroduced.   **5. Full Recovery (6-12 months):**   - **Return to sports:** Non-contact sports may resume around 4-6 months, but contact sports typically require 6-9 months for full return, depending on the surgeon's assessment and the patient's progress. - **Return to full activity:** Full recovery, including return to high-demand activities or contact sports, may take up to 9-12 months to ensure the shoulder is strong, stable, and ready for impact.   **Individual Factors:**   - Age, health status, the extent of the shoulder damage, and adherence to rehabilitation protocols play a critical role in recovery time. Following your surgeon’s advice and working closely with a physical therapist are essential to optimizing recovery.   If you’re considering surgery or are in recovery, a tailored rehabilitation plan can be developed based on your specific case. | This is for informational purposes only. For medical advice or diagnosis, consult a professional.  The recovery time after surgery for anterior shoulder instability can vary depending on the extent of the injury and the type of surgery performed. However, most people can expect to return to their normal activities within 6 months. ([ref](https://pmc.ncbi.nlm.nih.gov/articles/PMC10557935/#:~:text=Period%20of%20post%2Doperative%20immobilisation,between%201%20and%207%20weeks.))  Here is a general timeline for recovery:   - **0-6 weeks:** The arm will be in a sling to immobilize the shoulder. During this time, it is important to rest and avoid any activities that could put stress on the shoulder. ([ref](https://myhealth.alberta.ca/Health/aftercareinformation/pages/conditions.aspx?hwid=ug3937#:~:text=Your%20arm%20will%20also%20be,as%20long%20as%206%20weeks.)) - **6-12 weeks:** Physical therapy will begin to help regain range of motion and strength. - **12-24 weeks:** The focus will be on returning to normal activities. This may include light exercise and sports. - **6-12 months:** Full recovery is usually achieved within this time frame.   It is important to follow the instructions of your surgeon and physical therapist to ensure a successful recovery. |
| **When can I return to sports and drive after anterior shoulder instability treatment?** | The timeline for returning to sports and driving after treatment for anterior shoulder instability depends on several factors, including the type of treatment (surgical or non-surgical), the severity of the instability, and individual recovery progress. Here's a general guideline:  **1. Non-Surgical Treatment**   - **Physical Therapy**: If treated non-surgically with physical therapy to strengthen the muscles around the shoulder, most patients can return to non-contact sports within 6 to 12 weeks. However, contact sports or activities that stress the shoulder might take longer—usually 3 to 6 months—depending on the severity of the injury and response to therapy. - **Driving**: You can typically return to driving once you have regained sufficient range of motion and strength to safely control the vehicle. This is often around 2 to 4 weeks, but your doctor will need to assess your progress.   **2. Surgical Treatment (Shoulder Stabilization Surgery)**   - **Return to Sports**: After surgery, the timeline is generally longer due to the need for proper healing and rehabilitation:   - **Low-impact activities**: (like stationary cycling or walking) can resume after 6 weeks, but with restrictions on shoulder movement.   - **Non-contact sports**: Return is usually possible around 3 to 4 months.   - **Contact sports** (like rugby, football, or wrestling) or overhead sports (like tennis, volleyball, or swimming) may take **6 to 12 months** to avoid re-injury, depending on how well the shoulder heals and regains strength. - **Driving**: After shoulder surgery, most patients can return to driving once they have full functional use of their arm, which typically takes about 4 to 6 weeks. It depends on whether you are off pain medications and have the ability to control the steering wheel without pain or limitation.   **Final Considerations:**   - **Doctor's Clearance**: Always get clearance from your doctor or physical therapist before resuming sports or driving, as individual recovery can vary. - **Gradual Return**: Return to sports should be gradual, with modified activities that avoid stress on the shoulder initially, building up to full participation as strength, stability, and mobility improve.   Would you like more specific guidelines on certain sports or exercises? | The timeline for returning to sports and driving after **anterior shoulder instability treatment** depends on the treatment method (surgical vs. non-surgical) and individual recovery progress.  **1. Non-surgical treatment:**   - **Return to sports**: Typically, you can return to sports in **8–12 weeks** if your shoulder stabilizes well with physical therapy. However, this depends on the sport's intensity and shoulder involvement (e.g., contact sports may take longer). - **Driving**: You may be able to resume driving after **2–3 weeks**, once you can comfortably use your arm without pain and have regained a sufficient range of motion.   **2. Surgical treatment (e.g., arthroscopic stabilization or open surgery):**   - **Return to sports**: Usually, athletes can return to sports in **4–6 months**. High-risk or contact sports (football, rugby, wrestling) may require up to **6–9 months** to ensure the shoulder is fully stable and strong. - **Driving**: Driving is often permitted after **4–6 weeks**, once you regain functional range of motion and sufficient strength in your shoulder to safely control the steering wheel.   **Factors influencing recovery:**   - **Type of sport**: High-demand sports (like swimming or tennis) may need more time due to the shoulder's range of motion and strength demands. - **Shoulder strength and stability**: The focus on rehabilitation exercises will improve strength and stability, reducing reinjury risk. - **Surgeon's recommendations**: Post-operative protocols may vary, and it's important to follow your orthopedic surgeon's specific guidelines.   Always consult your orthopedic surgeon or physical therapist for personalized advice based on your recovery progress and treatment specifics. | This is for informational purposes only. For medical advice or diagnosis, consult a professional.  The timing of your return to sports and driving after anterior shoulder instability treatment will depend on several factors, including the type of treatment you received, the severity of your injury, and your individual progress in rehabilitation. It is important to follow the specific instructions of your surgeon and physical therapist regarding your return to activity.  Here are some general guidelines for returning to sports and driving after anterior shoulder instability treatment:  **Return to sports:**   - **Non-contact sports:** You may be able to return to non-contact sports, such as swimming or running, within a few weeks or months after surgery, once you have regained sufficient range of motion and strength. - **Contact sports:** It may take six months or more before you can return to contact sports, such as football or rugby, as these activities place a high demand on the shoulder joint.   **Driving:**   - You may be able to return to driving within a few weeks after surgery, once you have regained sufficient range of motion and strength to safely control the vehicle. However, it is important to avoid driving if you are taking any medications that could impair your ability to drive safely.   It is important to note that these are just general guidelines, and your individual recovery may vary. It is essential to consult with your surgeon and physical therapist to determine when it is safe for you to return to sports and driving.  **Here are some additional tips for returning to sports and driving after anterior shoulder instability treatment:**   - **Follow your surgeon's instructions:** Your surgeon will provide you with specific instructions regarding your return to activity. It is important to follow these instructions carefully. - **Progressive rehabilitation:** Your physical therapist will design a rehabilitation program to help you regain range of motion, strength, and stability in your shoulder joint. It is important to follow your rehabilitation program consistently and gradually increase the intensity of your exercises as you progress. - **Listen to your body:** If you experience any pain or discomfort, stop the activity and rest. It is important to avoid pushing yourself too hard, as this could lead to further injury. - **Return to activity gradually:** Once you have been cleared to return to activity, start slowly and gradually increase the intensity and duration of your workouts. ([ref](https://www.nhslanarkshire.scot.nhs.uk/services/physiotherapy-msk/shoulder-instability/#:~:text=You%20may%20be%20sore%20at,tasks%20that%20aggravate%20your%20symptoms.)) |
